# Flow Cytometry Immunophenotyping for Diagnostic Orientation and Classification of Pediatric Cancer Based on the EuroFlow Solid Tumor Orientation Tube (STOT)

**DOI:** 10.3390/cancers13194945

**Published:** 2021-09-30

**Authors:** Cristiane de Sá Ferreira-Facio, Vitor Botafogo, Patrícia Mello Ferrão, Maria Clara Canellas, Cristiane B. Milito, Sérgio Romano, Daiana V. Lopes, Lisandra C. Teixeira, Elen Oliveira, Enrico Bruno-Riscarolli, Fabiana V. Mello, Patrícia F. R. Siqueira, Patrícia Moura, Francisco Nicanor Macedo, Danielle N. Forny, Luíza Simião, Ana Luíza Pureza, Marcelo Gerardin Poirot Land, Carlos Eduardo Pedreira, Jacques J. M. van Dongen, Alberto Orfao, Elaine Sobral da Costa

**Affiliations:** 1 Internal Medicine Postgraduate Program, Faculty of Medicine, Federal University of Rio de Janeiro (UFRJ), Rio de Janeiro 21941-617, Brazil; cristianefacio@ippmg.ufrj.br (C.d.S.F.-F.); vbotafogo@usal.es (V.B.); lisandra_castro@hotmail.com (L.C.T.); elenoliveira@ippmg.ufrj.br (E.O.); enrico.riscarolli@edu.unirio.br (E.B.-R.); patriciasiqueira@ippmg.ufrj.br (P.F.R.S.); land.marcelo@ippmg.ufrj.br (M.G.P.L.); 2 Cytometry Service, Institute of Paediatrics and Puericultura Martagão Gesteira (IPPMG), Faculty of Medicine, Federal University of Rio de Janeiro (UFRJ), Rio de Janeiro 21941-612, Brazil; patferrao@gmail.com (P.M.F.); mariaclara.canellas@gmail.com (M.C.C.); daiana.alves@nupem.ufrj.br (D.V.L.); fabianamello@ippmg.ufrj.br (F.V.M.); luiza.simiao@yahoo.com.br (L.S.); analuiza.pureza@gmail.com (A.L.P.); 3 Department of Pathology, Faculty of Medicine, Clementino Fraga Filho University Hospital, Federal University of Rio de Janeiro (UFRJ), Rio de Janeiro 21941-617, Brazil; crismilito@medicina.ufrj.br; 4 Laboratory of Anatomical Pathology and Cytopathology, Instituto Nacional de Câncer (INCa), Rio de Janeiro 20220-400, Brazil; sergioromano@gmail.com; 5 I’Dor Institute, Hospital Estadual da Criança, Rio de Janeiro 21330-400, Brazil; patricia.moura@hcrianca.com.br (P.M.); nicanoramacedo@hotmail.com (F.N.M.); 6 Department of Pediatric Surgery, Institute of Paediatrics and Puericultura Martagão Gesteira (IPPMG), Faculty of Medicine, Federal University of Rio de Janeiro (UFRJ), Rio de Janeiro 21941-612, Brazil; dforny@ippmg.ufrj.br; 7 Systems and Computing Engineering Department (COPPE-PESC), Universidade Federal do Rio de Janeiro (UFRJ), Rio de Janeiro 21941-972, Brazil; pedreira@cos.ufrj.br; 8 Department of Immunohematology and Blood Transfusion (IHB), Leiden University Medical Center (LUMC), 2333 ZA Leiden, The Netherlands; J.J.M.van_Dongen@lumc.nl; 9 Translational and Clinical Research Program, Centro de Investigación del Cáncer and IBMCC (CSIC-University of Salamanca), Cytometry Service, NUCLEUS, Department of Medicine, University of Salamanca (USAL), Institute of Biomedical Research of Salamanca (IBSAL), 37007 Salamanca, Spain

**Keywords:** pediatric cancer, pediatric solid tumors, flow cytometry, diagnosis, standardization, immunophenotyping, STOT

## Abstract

**Simple Summary:**

Pediatric solid tumors are a heterogenous group of diseases that comprise ≈ 40% of all pediatric cancers, early diagnosis being key for improved survival. Here we designed, tested, and validated a single eight-color tube for the diagnostic screening of pediatric cancer—solid tumor orientation tube (STOT)—based on multiparameter flow cytometry vs. conventional diagnostic procedures. Prospective clinical validation of STOT in 149 samples (63 tumor mass, 38 bone marrow, 30 lymph node, and 18 body fluid samples) screened for pediatric cancer, apart from 26 blood specimens that were excluded from analysis, showed concordant results with the final WHO/ICCC-3 diagnosis in 138/149 cases (92.6%). This included correct diagnostic orientation by STOT in 43/44 (98%) malignant and 4/4 (100%) benign non-hematopoietic tumors, together with 28/38 (74%) leukemia/lymphoma cases. The only recurrently missed diagnosis was Hodgkin lymphoma (0/8), which would require additional markers. These results support the use of STOT as a complementary tool for fast and accurate diagnostic screening, orientation, and classification of pediatric cancer in suspicious patients.

**Abstract:**

Early diagnosis of pediatric cancer is key for adequate patient management and improved outcome. Although multiparameter flow cytometry (MFC) has proven of great utility in the diagnosis and classification of hematologic malignancies, its application to non-hematopoietic pediatric tumors remains limited. Here we designed and prospectively validated a new single eight-color antibody combination—solid tumor orientation tube, STOT—for diagnostic screening of pediatric cancer by MFC. A total of 476 samples (139 tumor mass, 138 bone marrow, 86 lymph node, 58 peripheral blood, and 55 other body fluid samples) from 296 patients with diagnostic suspicion of pediatric cancer were analyzed by MFC vs. conventional diagnostic procedures. STOT was designed after several design–test–evaluate–redesign cycles based on a large panel of monoclonal antibody combinations tested on 301 samples. In its final version, STOT consists of a single 8-color/12-marker antibody combination (CD99-CD8/_nu_myogenin/CD4-EpCAM/CD56/GD2/_sm_CD3-CD19/_cy_CD3-CD271/CD45). Prospective validation of STOT in 149 samples showed concordant results with the patient WHO/ICCC-3 diagnosis in 138/149 cases (92.6%). These included: 63/63 (100%) reactive/disease-free samples, 43/44 (98%) malignant and 4/4 (100%) benign non-hematopoietic tumors together with 28/38 (74%) leukemia/lymphoma cases; the only exception was Hodgkin lymphoma that required additional markers to be stained. In addition, STOT allowed accurate discrimination among the four most common subtypes of malignant CD45^−^ CD56^++^ non-hematopoietic solid tumors: 13/13 (GD2^++^ _nu_myogenin^−^ CD271^−/+^ _nu_MyoD1^−^ CD99^−^ EpCAM^−^) neuroblastoma samples, 5/5 (GD2^−^ _nu_myogenin^++^ CD271^++^ _nu_MyoD1^++^ CD99^−/+^ EpCAM^−^) rhabdomyosarcomas, 2/2 (GD2^−/+^ _nu_myogenin^−^ CD271^+^ _nu_MyoD1^−^ CD99^+^ EpCAM^−^) Ewing sarcoma family of tumors, and 7/7 (GD2^−^ _nu_myogenin^−^ CD271^+^ _nu_MyoD1^−^ CD99^−^ EpCAM^+^) Wilms tumors. In summary, here we designed and validated a new standardized antibody combination and MFC assay for diagnostic screening of pediatric solid tumors that might contribute to fast and accurate diagnostic orientation and classification of pediatric cancer in routine clinical practice.

## 1. Introduction

Approximately 300,000 new cancer cases are diagnosed worldwide in children and adolescents below the age of 19 every year, with an annual rate of around 80,000 deaths [1]. Of these patients, around half correspond to leukemia (28%) and lymphoma (19%) cases, while the other half correspond to central nervous system tumors (27%) and a broad variety of extracranial non-hematopoietic pediatric cancer types (26%) [1]. Early diagnosis and fast (accurate) classification of pediatric cancers are key for guiding optimal therapeutic choice and for adequate patient management [2,3].

At present, diagnosis and classification of pediatric non-hematopoietic (solid) tumors are guided by the clinical manifestations of the disease, imaging, and laboratory findings, and they are subsequently confirmed, in virtually all cases, via invasive (e.g., tissue biopsy) procedures based on conventional histopathology, immunohistochemistry (IHC), and cytogenetic/molecular analysis of tumor tissues [2]. Based on this diagnostic approach, the great majority (≈80%) of all extracranial pediatric non-hematopoietic tumors are morphologically classified as small round cell tumors (SRCT) which (frequently) originate from immature cells resembling embryonic cells from different tissues [4]. Thus, extensive IHC panels of markers are subsequently applied for further diagnostic classification of SRCT according to the cell lineage and maturation stage of the tumor cells. Such antibody panels aim at the identification of the tissue origin of the tumor and include reagents for myogenic (e.g., _nu_MyoD1, _nu_myogenin, desmin, myoglobin), neural/neural crest (e.g., NSE, NB-84, TrkA, NFTP, synaptophysin, CD56, CD99), and germ cell tissues (e.g., alpha fetoprotein, PLAP, cytokeratin), in addition to mesenchymal (e.g., vimentin, smooth muscle actin) and hematopoietic cell-associated markers (e.g., CD45, CD3, CD19, CD20, myeloperoxidase (MPO)) [2,5,6]. Despite all these markers, in many patients, diagnosis and classification of the underlying pediatric non-hematopoietic tumor is delayed or even remains a challenge due to the small and paucicellular nature of biopsy samples, the morphological similarities, and the overlapping immunohistochemical features and immunophenotypes observed by optical microscopy among distinct types of SRCT [5,7].

Multiparameter flow cytometry (MFC) is a key technique for the immunophenotypic diagnosis of acute leukemias and chronic lymphoproliferative disorders, with the ability to provide data on simultaneous evaluation of multiple proteins in hundred thousand to millions of single cells [8]. Despite this, MFC is not part of the routine diagnostic workup of pediatric solid tumors [9,10,11]. This is mostly due to the need of (fresh) single cell suspensions and the fact that unlike IHC, MFC does not provide information on the structure and tissue location of tumor cells [9]. Thus, early MFC reports in pediatric solid tumors have mainly focused on the identification of disseminated disease in bone marrow (BM) and/or blood [12,13,14]. Interestingly, these studies already revealed different antigen expression profiles among BM metastatic non-hematopoietic tumor cells, some of which emerged as strongly associated with (or even specific for) some diagnostic subtypes of pediatric solid tumors [15,16,17,18]. Thus, co-expression of CD56^+^, CD90^+^, GD2^+^, CD81^+^, and CD9^+^ in the absence of CD45 is a typical immunophenotypic feature of neuroblastoma (NBL) cells [19,20], while a CD99^+^ CD45^−^ phenotype has been associated with Ewing sarcoma (ES) [14,18], and co-expression of CD90^+^, CD56^+^, and CD57^−/+^ in the absence of CD45 is most frequently observed in rhabdomyosarcoma (RMS) tumor cells [16,21].

Despite promising data provided by these MFC reports, to our knowledge, no serious attempt has been made so far to design and validate a combination of markers for fast MFC diagnostic screening, orientation, and classification of non-hematopoietic pediatric tumors. In this regard, in 2013, we evaluated the (potential) clinical utility of a comprehensive MFC panel of 32 markers for the diagnostic orientation and classification of pediatric solid tumors, based on a limited series of 52 samples from 40 patients suspicious of pediatric cancer [22]. The panel of markers evaluated in this preliminary study showed high diagnostic accuracy (96%) vs. conventional histopathology [22], supporting the great potential of MFC to detect tumor cells in pediatric non-Hodgkin (NHL) [23,24] and Hodgkin lymphoma (HL) [25], RMS [26], NBL [26,27], and carcinomas [28,29]. In turn, this study also revealed the limited utility and potential redundancy of several markers, together with the need for additional stainings for improved diagnosis of several specific tumor subtypes [22], that should be combined into a single multicolor antibody combination for screening purposes.

Here we designed, tested, and validated an 8-color/12-marker antibody combination for diagnostic screening, orientation, and classification of pediatric cancer (i.e., solid tumor orientation tube, STOT) by MFC, which allows simultaneous identification, enumeration, and characterization of hematopoietic vs. non-hematopoietic tumor cells and tissue resident and infiltrating immune cells.

## 2. Results

### 2.1. Distribution of Tumor Subtypes per Type of Sample

Tumor cells from a total of 36 different WHO/ICCC-3 diagnostic categories of pediatric solid tumors were detected in 22 different types of specimens for the 476 samples investigated (Table 1). Tumor masses were the most frequent type of specimen received from children with suspicion of solid tumor (139/476, 29%). In addition, tumor mass specimens—116/139 (83%) corresponding to pediatric cancer patients, with only 2/116 (2%) corresponding to disease-free samples (1 contralateral testis biopsy without B-cell leukemic infiltration and 1 non-infiltrated tumor border of a Wilms tumor (WT) patient); 8/139 (6%) corresponding to benign pediatric tumors, and 15/139 (11%) reactive cases—were also the most frequent type of sample infiltrated by malignant cells (114/139, 83%). From the tumor mass specimens, those corresponding to patients diagnosed with non-hematopoietic solid tumors (82/139, 59% corresponding to 18 distinct WHO/ICCC-3 diagnostic subtypes) were the most common infiltrated samples (81/82, 99%), followed by those of patients with hematopoietic malignancies (34/139, 24% cases corresponding to 11 distinct WHO diagnostic categories) with 33/34 samples (97%) infiltrated by malignant hematopoietic cells.

Among all tumor mass specimens from children diagnosed with a solid tumor, abdominal tumor masses (*n* = 58) were those more frequently infiltrated by pediatric cancer (48/58, 83%), with only 5 (9%) reactive/disease-free samples and 5 (9%) samples of benign tumors (mesoblastic nephroma, 2; vascular tumors, 2; and cystic nephroma, 1). Within all tumor mass specimens, abdominal tumor masses showed the highest variety of non-hematopoietic WHO/ICCC-3 diagnostic subtypes of pediatric cancer (*n* = 11), including: 43 non-hematopoietic neoplasms (18 NBL, 13 WT, 1 germ cell tumor (GCT), 1 extraosseous Ewing sarcoma (EES), 1 clear cell sarcoma, 2 hepatoblastomas, 1 renal carcinoma (RC), 1 Frantz tumor, 1 pheochromocytoma (PHEO), and 4 adrenal carcinomas) and 5 Burkitt lymphoma (BL) samples.

The second most frequently studied type of specimen was BM (138/476 samples, 29%) with a significantly lower rate of infiltration (*p* = 0.05 vs. tumor masses) by neoplastic cells (41/138, 30%). BM infiltrating tumors mostly corresponded to hematopoietic tumors (23/41, 56%), NBL (15/41, 37%), and, less frequently, to metastatic RMS (3/41, 7%). In turn, lymph node specimens (86/476 samples, 18%) were found to be infiltrated by neoplastic cells in over one third of the cases (32/86, 37%). Similarly, almost half of all body fluid samples other than blood that were investigated (28/55, 51%), were found to be infiltrated by neoplastic cells, most of which corresponded to hematological malignancies (23/28, 82%) and a smaller fraction to non-hematopoietic cancer (5/28, 18%). Finally, 13/58 (22%) blood samples analyzed had detectable circulating tumor cells corresponding to 4 non-hematopoietic cancers (3 NBL and 1 RMS) and 9 hematopoietic tumors (1 anaplastic large cell lymphoma (ALCL), 1 B-cell precursor acute lymphoblastic leukemia/lymphoma (BCP-ALL), 3 T-cell lymphoblastic lymphoma (T-LL), 1 BL, 1 Epstein-Barr virus (EBV)-related lymphoma and 2 acute myeloid leukemia (AML)) (Table 1).

### 2.2. Cell Viability per Type of Sample

Overall, a median cell viability of 59% (0.7−98%) was observed after excluding events corresponding to dead cells and cell debris, with viability rates > 50% in two thirds of the samples (322/476, 68%) (Table 1). The three different protocols (PI, DRAQ5, and Boolean gating strategy) used to access cell viability yielded similar results for the percentage of viable cells: 60% (±12%) and 54% (±19%) with PI and light scatter gating approaches, respectively, vs. 44.5% (±34%) with the DRAQ5-based method. Therefore, the light scatter based Boolean gating strategy was chosen for viability assessment in subsequent specimens.

Based on this approach, tumor mass specimens (225/476, 47%) showed the lowest median (range) cell viability, 45% (0.7–93%), whereas blood, BM, and other body fluid samples were those that displayed the highest median (range) cell viability: 75% (20%–97%), 80% (13%−97%), and 72% (2%–98%), respectively. Of note, only 3% (15/476) of samples had cell viabilities of <5%, which was almost systematically due to delayed (>12–24 h) sample transportation and delivery to the central MFC laboratory. Despite the low cell viability of such samples, neoplastic cells were still clearly identified by MFC in 13/15 (87%) samples. However, decreased cell viability hampered accurate quantification of tumor cells in these samples (vs. IHC/cytochemistry).

### 2.3. Selection of Backbone Markers to Discriminate between Normal/Reactive and Neoplastic Cells

**Figure 1:**
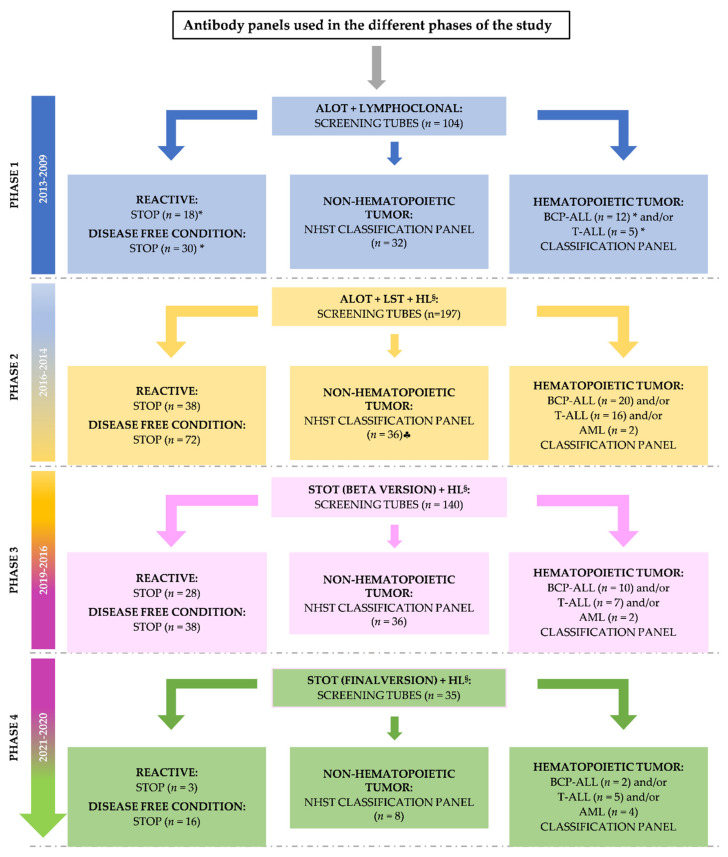
Flowchart of the distinct phases of the study and the corresponding antibody panels evaluated for the diagnostic screening, orientation, and classification of pediatric tumors. * Among these samples, 4 reactive samples, 11 disease-free samples, and 7 hematopoietic malignant samples were also tested with the non-hematopoietic solid tumor (NHST) panel. ♣ In addition to these samples, 6 samples the NHST complete panel could not be stained because of very limited cellularity. ^§^ The Hodgkin lymphoma (HL) tube was stained in 72 samples across the 3 phases in which it was tested, including specimens from 7 ALCL, 15 HL, 7 hematopoietic tumors, 10 non-hematopoietic tumors, and 33 reactive/disease free lymph node samples. Abbreviations: ALOT—acute leukemia orientation tube; AML—acute myeloid leukemia; BCP-ALL—B-cell precursor–acute lymphoblastic leukemia; LST—lymphoid screening tube; T-ALL—T-cell acute lymphoblastic leukemia; STOP–end of investigation.

**Figure 2:**
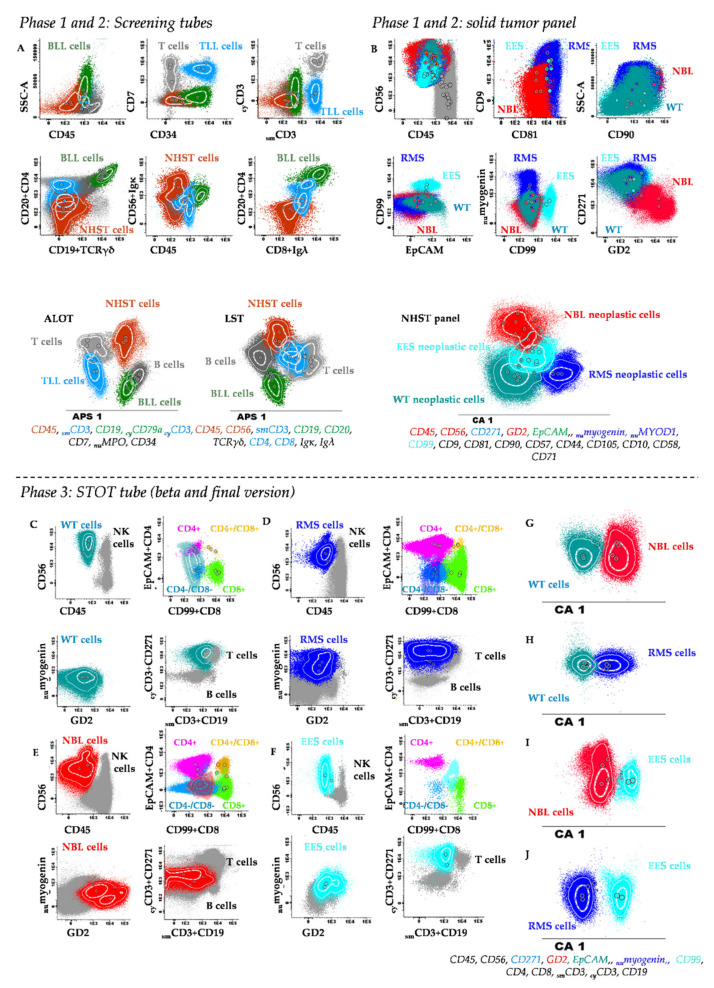
Flow cytometric identification and characterization of normal/reactive immune cells vs. hematopoietic and non-hematopoietic tumor cells with the antibody panel used in phases 1 and 2 (**A**,**B**) and in phase 3 (**C**–**J**) of the study. (**A**) Conventional bivariate dot plots and APS diagrams showing merged data files containing distinct subtypes of CD45^−^ CD56^+^ non-hematopoietic solid tumor cells (NHST) (dark red events), B lymphoblastic lymphoma cells (BLL) (green events), T lymphoblastic lymphoma cells (TLL) (blue events), and normal immune cells (grey events) from solid tumor samples stained with the screening tubes. (**B**) Solid tumor antibody panel used in phase 1 and 2 of the study, showing tumor cells of distinct NHST subtypes—neuroblastoma (NBL) (red dots), rhabdomyosarcoma (RMS) (dark blue dots), extraosseous Ewing sarcoma (EES) (light blue dots), Wilms tumor (WT) (petrol blue dots). In panels A and B, the most informative markers for identification of tumor cells and their subclassification with both sets of antibody combinations are shown in red, blue, and green at the bottom of the corresponding APS diagram. (**C**–**J**) Samples stained with STOT in phase 3 of the study are shown. Bivariate dot plots of distinct combination of the STOT markers for discrimination between residual immune cells (colored grey, pink, light green, and/or yellow) and tumor cells contained in merged data files of WT (petrol blue dots), RMS (dark blue dots), NBL (red dots) and EES (light blue dots) are shown in plots (**C**–**F**), respectively. Multivariate canonical (CA) analysis of the overall 1 × 1 comparisons between the phenotype of: (**G**) WT (petrol blue dots) vs. NBL (red dots); (**H**) WT (petrol blue dots) vs. RMS (dark blue dots); (**I**) NBL (red dots) vs. EES (light blue dots); and (**J**) RMS (dark blue dots) vs. EES (light blue dots) are shown in panels G to J, respectively. At the bottom of these later plots, the most informative STOT markers discriminating the above tumor subtypes are color-coded.

In the first design–test–redesign phase of the study (*n* = 104 samples), we aimed at identifying normal (tissue) cells and discriminating them from infiltrating hematopoietic and non-hematopoietic (solid) tumor cells, based on the ALOT and Lymphoclonal^TM^ screening tubes (Figure 1). Thus, residual normal/reactive immune cells were identified in all 104 samples investigated, and they consisted of lymphocytes (CD45^++^ FSC^lo^ SSC^lo^) and their major populations of B-cells (CD19^+^ _cy_CD79a^+^) and their Igλ^+^ and Igκ^+^ subsets, plasma cells (CD19^+^ CD45^lo^ CD38^++^), NK-cells (CD56^+^ CD7^+^ _sm_CD3^−^ _cy_CD3^−/+^), T-cells (CD7^+^ _cy_CD3^+^ _sm_CD3^+^) and their TCD4^+^/CD8^−^, TCD4^−^/CD8^+^, TCD4^−^/CD8^−^, and TCD4^+^/CD8^+^ subsets, in addition to monocytes (CD45^++^ CD4^+^ CD38^+^ MPO^−/lo^ FSC^int^ SSC^int^) and neutrophils (CD45^+^ MPO^+^ CD10^+^ FSC^lo/int^ SSC^hi^) (Figure 2). Suspicious CD45^−^ non-hematopoietic malignant tumor cells were also identified by MFC in 30/104 (29%) samples investigated. In most samples infiltrated by non-hematopoietic malignant tumor cells (25/30, 83%), suspicious CD45^−^ tumor cells were CD56^+^ and lacked expression of all hematopoietic markers tested with ALOT and Lymphoclonal^TM^ (_sm_CD3, _cy_CD3, CD19, CD5, CD20, Igκ, Igλ, CD34, CD7, CD4, CD8, _cy_CD79a, and _nu_MPO), except for 2 benign non-hematopoietic vascular tumors (1 hemangiopericytoma and 1 hemangioma) in which tumor cells co-expressed CD34^+^. In turn, in 17/23 (71%) leukemia/lymphoma cases, hematopoietic tumor cells were identified, including 14/17 (82%) CD45^+^ leukemias/lymphomas and 3/17 (18%) CD19^+^ _cy_CD79a^+^ CD45^−^ B-LL samples (Figure 2). No tumor cells were identified in any of the HL (0/5) and ALCL (0/1) infiltrated (lymph node) samples with the ALOT and Lymphoclonal^TM^ screening tubes used in this first phase.

Subsequently, all (gated) MFC data files generated in this first phase, except for those corresponding to the ALCL and HL samples, were merged into a single data file. Multivariate analysis (i.e., PCA) of the merged flow cytometry data was then performed to select the most informative, non-redundant markers to discriminate between non-hematopoietic tumor cells, leukemia/lymphoma cells and normal residual immune/hematopoietic cells. Thus, PCA revealed that: i) CD45 and CD56 were the most informative *backbone markers* for distinction between CD45^−^ non-hematopoietic tumor cells and CD45^+^ normal cells, whereas ii) CD19, _cy_CD3, and _sm_CD3 in combination with CD45 were critical to distinguish between leukemia/lymphoma cells and non-hematopoietic tumor cells (Figure 2). Based on these findings, CD45, CD56, CD19, _cy_CD3, and _sm_CD3 were selected from all markers evaluated, as the minimum combination of backbone markers for identification of non-hematopoietic and hematopoietic tumor cells (except in ALCL and HL infiltrated samples). In samples showing non-hematopoietic tumor cells as identified with the screening tubes, further disease classification could not be achieved without the markers included in the additional non-hematopoietic solid tumor panel (Figure 1 and Table 2). However, with this later panel, accurate diagnostic orientation was achieved in 32/32 (100%) non-hematopoietic tumor cases (of 11 distinct tumor types) in the first phase of panel design.

Based on the results obtained in this first phase of the study, a third HL screening tube was designed for improved detection of HL and ALCL tumor cells in (suspicious) lymph node samples in subsequent phases 2 and 3 of the study. In addition, in this second phase, the Lymphoclonal^TM^ tube was replaced by LST, which included both CD45 and CD56 for improved distinction between hematopoietic and non-hematopoietic tumors and, e.g., CD4, CD8, anti-Igκλ, and CD38 for more accurate B, T, and NK cell subsetting (Table 2). As a consequence of these modifications, from the second phase of the study onwards, the use of the ALOT and LST screening tubes together with the new HL tube was associated with a significantly increased efficiency in the differential diagnosis between reactive/disease-free lymph node samples (12/12, 100%) and both ALCL (8/8, 100%) and HL (8/15, 53%). Of note, in the 7/15 HL infiltrated samples by histopathology/IHC in which HL cells were not detected by MFC, <10^6^ cells (range: 8 × 10^4^–7 × 10^5^ cells) had been evaluated by MFC (vs. 2.5 × 10^5^–5.3 × 10^6^ cells in the 8/15 HL cases correctly identified by MFC).

### 2.4. Immunophenotype of Tumor Cells from Distinct WHO/ICCC-3 Diagnostic Categories of Non-Hematopoietic Neoplasms

The immunophenotype of non-hematopoietic tumor cells identified in 118 infiltrated samples (44 neuroblastic tumors, 17 kidney tumors, 2 liver tumors, 4 malignant bone tumors, 20 soft tissue tumors, 13 germ cell tumors, 7 malignant epithelial tumors, and 3 other rare neoplasms, in addition to 8 benign tumors), plus that of cells from 7 leukemia/lymphoma infiltrated samples, 6 reactive samples and 7 disease-free samples was investigated with the solid tumor antibody panel for the GD2, CD271, EpCAM, _nu_myogenin, _nu_MyoD1, CD99, CD90, CD81, CD9, CD10, CD44, CD57, CD58, CD71, CD105, and CD34 markers (Table 2). This dataset was then used to select those markers that, apart from CD45 and CD56, would most contribute to sub-classification of non-hematopoietic tumor cells into the most common diagnostic categories of non-hematopoietic small round cell tumors (SRCT) (e.g., NBL, RMS, EWS, and WT) (Table 2 and Table S1).

**Figure 3:**
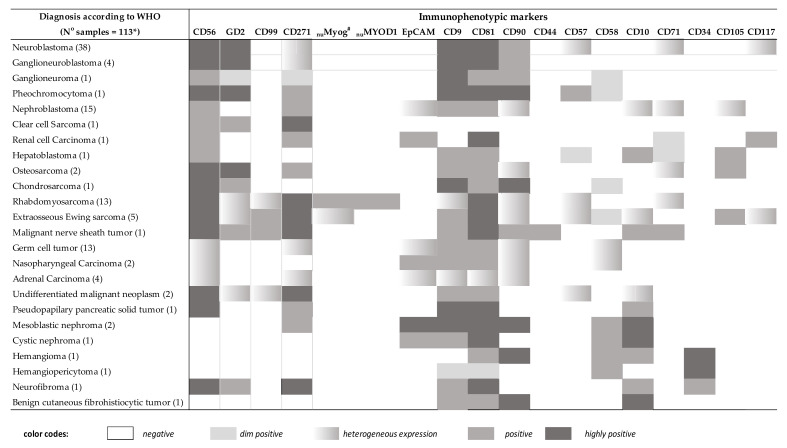
Pattern of expression of individual immunophenotypic markers in distinct WHO/ICCC-3 diagnostic categories of pediatric solid tumors. Heat map summarizing the intensity and pattern of expression of different phenotypic markers for the different diagnostic subtypes of pediatric solid tumors based on both the mean fluorescent intensity level of expression per/cell and the percentage of positive cases. * 5 samples were not diagnosed by MFC (1 hepatoblastoma, 1 osteosarcoma, 1 extraosseous Ewing sarcoma, 1 nasopharyngeal carcinoma, 1 hemangioma). # In one patient with an Ewing sarcoma, _nu_myogenin expression was positive both on MFC and IHC.

From the phenotypic point of view, tumor cells from all neuroblastic tumors investigated (38 NBL, 4 ganglioneuroblastomas, 1 ganglioneuroma, and 1 pheochromocytoma) except 1 (43/44, 98%) showed a rather homogenous phenotype with systematic positivity for CD56^++^, GD2^++^, CD90^+^, CD9^++^, and CD81^++^ while they lacked expression of CD99, EpCAM, CD10, _nu_MyoD1, and _nu_myogenin. Other markers expressed in tumor cells from a minority of neuroblastic tumor samples included: CD57 positive in 5/43 samples (11.6%), CD271 expressed at low levels in 7/43 samples (16%), and CD117 that was positive in 4/43 samples (9.3%). Of note, among neuroblastic tumors, GD2 expression was significantly lower in NBL cells from patients with localized (14/43) vs. metastatic NBL (29/43)—median (range) fluorescence intensity (MFI) of 3053 (1309–38,704) vs. 18,530 (3227–92,369); *p* = 0.005. In turn, CD271^lo^ cases corresponded to 4/38 neuroblastomas, 1/2 ganglioneuromas, and 2/4 ganglioneuroblastoma cases, while CD271 was systematically negative in all 29 metastatic NBL cases. The only pheochromocytoma analyzed (*n* = 1) showed a distinct CD56^++^ CD57^+^ GD2^++^ CD271^+^ CD9^+^ CD81^+^ CD90^het^ CD58^+^ phenotype, in the absence of expression of CD10, CD99, CD117, EpCAM, _nu_MyoD1, and _nu_myogenin. In Figure 3 and Table S2, a graphical summary of these phenotypic profiles is provided in comparison with other non-hematopoietic cancer types described below.

In contrast to neuroblastic tumors, soft tissue tumors and other extraosseous sarcomas—*n* = 20 (18%), with 19/20 infiltrated by MFC—had a common CD56^++^ CD271^++^ CD81^++^ phenotype in the absence of CD45, CD34, and EpCAM expression, with three distinct (heterogeneous) phenotypic profiles (Figure 3 and Table S2). Thus, in 5/5 infiltrated EES samples, tumor cells displayed a CD99^+^ CD9^+^ _nu_MyoD1^−^ phenotype with expression of GD2^lo^ in 2/5 (40%), CD117^+^ in 2/5 (40%), CD90^+^ in 4/5 (80%), CD57^+^ CD10^+^ in 1/5 samples (20%), CD105^+^ in 2/2 (100%) of samples tested and dim expression of _nu_myogenin in association with coexisting EWS and PAX3 gene rearrangements in 1/5 EES tumors. In contrast to EES, tumor cells from 13/13 RMS cases were positive for _nu_MyoD1 and/or _nu_myogenin, in the absence of CD10, CD105, and CD58 expression, showed variable percentages of CD90^+^ (5/13, 39%) and CD9^+^ (5/13, 39%) and heterogeneous levels of expression of GD2, CD57, and CD99 in 1/13 (7%) RMS case (Figure 3 and Table S2). The remaining sarcoma tumor corresponded to a malignant nerve sheath tumor, which displayed a CD56^+^ CD99^+^ GD2^+^ CD271^+^ CD9^+^ CD81^+^ CD90^+^ CD44^+^ CD105^−^ CD10^+^ CD71^+^ phenotype, in the absence of expression of all other markers tested.

Malignant bone tumors represented a small subgroup of all solid tumors investigated (4/110 samples, 3.6%) and showed a heterogenous phenotype. Thus, GD2, CD9, CD105, and CD71 were positive in two osteosarcomas (OS), while CD90 was expressed in only one case, always in the absence of _nu_MyoD1, _nu_myogenin, CD57, and CD99 expression. In turn, tumor cells from the only chondrosarcoma (CDS) analyzed were also positive for GD2 and CD9, in addition to CD81 and CD90, and they lacked CD10, CD58, _nu_MyoD1, _nu_myogenin, CD57, and CD99 expression (Figure 3 and Table 2). In the remaining malignant bone tumor case, tumor cells were not detected by MFC (potentially) due to low (i.e., 1%) cell viability.

Tumor cells from all 17 malignant kidney neoplasms studied showed expression of CD271^+^ and CD81^hi^, together with positivity for EpCAM in the majority (12/17) of tumors (70%) and CD90 in 5/17 (29%), while they systematically lacked CD57, CD58, CD99, CD117, _nu_MyoD1, and _nu_myogenin expression (Figure 3 and Table S2). This phenotype was clearly distinct from that of the three benign kidney tumors (one cystic nephroma and two mesoblastic nephromas) studied, in which non-hematopoietic (benign) tumor cells were negative for both CD45 and CD56, while they co-expressed HLA-DR, CD10, and EpCAM. Interestingly, CD90 and CD271 were positive only on the mesoblastic nephromas (*n* = 2) but not on the cystic nephroma analyzed. In turn, in the 15 WT (out of 17 malignant kidney tumors analyzed) samples, neoplastic cells co-expressed CD9, CD81, and CD271 in the absence of HLADR and GD2, usually in the context of an EpCAM^+^ (13/15, 86%) phenotype. Furthermore, CD90 was expressed in 5/15 WT (33%), while CD10 was positive in only 3/15 WT cases (20%). Other less prevalent malignant kidney tumors included a renal cell carcinoma (RC), which showed a CD271^+^ CD81^+^ EpCAM^+^ CD71^+^ CD117^+^ phenotype in the absence of CD9, CD10, CD58, CD90, CD105, and GD2, and a clear cell sarcoma, which expressed CD271^+^ and GD2^+^ without EpCAM (Figure 3 and Table S2).

All GCT analyzed (*n* = 13) were positive for CD81 and CD9, while negative for CD10, CD99, CD58, GD2, _nu_MyoD1, _nu_myogenin, and other lineage-associated markers. A few additional markers were expressed only in a fraction of cases: CD56 was found in 5/13 tumors (39%), CD271 in 5/13 tumors (39%), CD90 in 5/13 tumors (39%) and EpCAM in 4/13 tumors (30%) (Figure 3 and Table S2). In turn, among malignant epithelial tumors (*n* = 7), the four adrenal carcinomas were heterogeneously positive for CD56, EpCAM, CD90, CD9, CD81, and CD271, while they were negative for GD2, CD99, _nu_MyoD1, _nu_myogenin, CD57, CD10, and CD58; neoplastic cells in 2/3 nasopharyngeal carcinomas expressed EpCAM, CD81, and CD9 in the absence of GD2, _nu_MyoD1, _nu_myogenin, CD90, CD271, and CD99, one case being CD56^+^ and the other CD56^−^ (Figure 3 and Table S2). In the third nasopharyngeal carcinoma and in one in two hepatoblastoma cases, tumor cells were not identified, potentially because of decreased cell viability (30% and 7%, respectively). The other hepatoblastoma tumor showed positivity for CD10, CD9, CD81, CD105 in the absence of GD2, EpCAM, CD99, _nu_myogenin, and CD90. The remaining three malignant tumors corresponded to rare neoplasms and included i) one CD56^++^ CD271^+^ CD9^++^ CD81^++^ CD10^+^ pseudopapillary tumor of the pancreas (Frantz tumor) (*n* = 1) and ii) two CD56^++^ CD81^+^ CD90^−^ CD271^++^ undifferentiated malignant tumors that could not be further classified by histopathology and IHC; in one of these two later tumors, neoplastic cells co-expressed CD99^+^ and GD2^+^, while in the other sample, they were CD99^−^ GD2^−^ CD10^+^ CD57^+^ CD9^+^ _nu_MyoD1^−^ _nu_myogenin^−^.

In parallel to the 110 malignant tumors identified, 8 benign tumor samples were also identified by MFC, consisting of 2 mesoblastic nephromas, 1 cystic nephroma (described above together with the malignant kidney tumors), 2 (benign) vascular tumors (1 hemangiomas and 1 hemangiopericytoma) that showed a unique phenotype with high co-expression of CD34 and CD81 (in addition to either CD90 and CD10 in the hemangioma or to CD9 and CD58 in the hemangiopericytoma), one unclassifiable tumor due to low cell viability, 1 CD56^++^ CD271^++^ CD9^+^ CD81^++^ CD10^+^ CD34^+^ neurofibroma and 1 CD10^++^ CD90^++^ CD9^+^ CD81^+^ CD99^−^ CD271^−^ CD57^−^ EpCAM^−^ benign cutaneous fibrohistiocytic tumor.

### 2.5. Design of the Solid Tumor Orientation Tube (STOT)

In order to select the most informative markers for the differential diagnosis among distinct WHO/ICCC-3 diagnostic categories of pediatric (non-hematopoietic) solid tumors, a single MFC data file was created by merging phenotypic data of (gated) non-hematopoietic cells from each tumor into a single data file. Subsequently, comparison among immunophenotypic data for each pair of diagnostic categories of non-hematopoietic tumor cells contained in the merge data file was performed, using (multivariate) PCA (Figure 1 and Figure 2). Thus, comparison of the four most frequent diagnostic subtypes of SRCT (i.e., RMS, EES, WT, and NBL) revealed that the most informative (non-redundant) markers for the differential diagnosis among those four entities were: i) CD99, EpCAM, _nu_myogenin, and CD271 for the EES vs. WT comparison; ii) CD9, CD99, CD271, and GD2 to discriminate EES from RMS; iii) CD271, GD2, CD99, and CD81 for differentiating EES from NBL; iv) CD271, CD9, _nu_myogenin, and CD81 to distinguish WT from RMS; v) GD2, CD271, EpCAM, and CD56 for the distinction between WT and NBL; and, vi) CD271, GD2, CD56, and CD99, for the RMS vs. NBL comparison (Figure 2 and Figure 3). Afterward, we evaluated those MFC markers that most contributed to differentiate distinct tumor subtypes inside each of the above WHO/ICCC-3 categories. Thus, among neuroblastic tumors, i) CD90, GD2, CD9, and CD56 were the most discriminative between NBL and GNB, whereas ii) CD271, EpCAM, CD9, and CD81 discriminated NBL from PHEO. Among soft tissue sarcomas, CD271, GD2, CD9, and CD90 allowed for the differential diagnosis between EES and RMS, and the CD9, CD81, CD99, and CD271 markers distinguished between OS and CDS. Finally, GD2, CD271, EpCAM, and CD99 discriminated between WT and CCS; CD81, CD9, CD99, and EpCAM distinguished WT from RC; and CD271, EpCAM, GD2, and CD99 allowed the differential diagnosis between RC and CCS.

Based on the results above, CD99, _nu_myogenin, EpCAM, GD2, and CD271 were identified as the most informative set of markers for the differential diagnosis among the most frequent diagnostic subtypes of non-hematopoietic malignant tumors. Thereby, these five markers were selected to be included in the first (β) version of STOT, in addition to the previously selected CD56, CD45, _sm_CD3, _cy_CD3, CD19, CD4, and CD8 (hematopoietic cell) markers (Table 2).

### 2.6. Prospective Validation of the Solid Tumor Orientation Tube (STOT) against the WHO/ICCC-3 Diagnosis

The performance of STOT (β version and final version) for the diagnostic screening and orientation of pediatric cancer was prospectively validated in 149/175 samples (after excluding 26 blood samples) from patients suspicious of non-hematopoietic cancer, against the (pre-defined) WHO/ICCC-3 diagnosis (Table 3). Overall, a high degree of agreement was observed in 138/149 samples (92.6%). Per diagnostic category, 100% agreement was observed in 31/31 reactive, 32/32 tumor-free samples, and in 4/4 benign tumors. Similarly, non-hematopoietic malignant tumor cells were detected in 43/44 samples that were infiltrated by histopathological findings (98% agreement), and in 28/38 (74%) leukemia/lymphoma samples (Table 3). The only discrepant (osteosarcoma) case showed low (1%) cell viability in the absence of detectable tumor cells by MFC.

Among the 44 (malignant) non-hematopoietic tumors analyzed at this stage, STOT was sufficient to reach the final WHO/ICCC-3 diagnosis in 27/44 (61.3%) samples, including all NBL (13/13), RMS (5/5), EES (2/2), and WT (7/7) samples. However, for the diagnostic classification of other less prevalent non-hematopoietic pediatric tumor subtypes (six GCT, three osteosarcomas, three adrenal carcinomas, one malignant nerve sheath tumor, one clear cell sarcoma, one undifferentiated malignant neoplasm, one pseudopapillary pancreatic solid tumor, and one hepatoblastoma), additional markers included in the non-hematopoietic tumor cell panel were required. Although only four benign tumors (a cystic nephroma, a mesoblastic nephroma, a hemangioma, and a neurofibroma) were studied, benign CD45^−^ and CD56^−^ tumor cells were detected with STOT in all four samples. Similarly, additional markers to those included in STOT were required for further subclassification of these four benign tumors, in which final diagnosis was reached based on positivity for i) CD34 in both of the two hemangioma tumors, ii) CD10 and HLADR in one benign kidney tumor, and iii) CD10^+^ CD9^+^ CD81^+^ CD105^+^ on a neurofibroma tumor; of note, the phenotypic profiles of those four benign tumors were clearly distinct from those found among malignant tumor cells.

Regarding hematopoietic (pediatric) tumors, STOT properly identified and classified tumor cells in 28/38 (74%) leukemia/lymphoma samples, with accurate orientation towards the B-, T- and/or myeloid cell lineage in 28/28 (100%) samples. However, the additional markers included in the EuroFlow classification panels [10] (Table S4) were required for final diagnosis in all 28 samples. Of note, failure of STOT mostly corresponded to HL samples (0/8 samples) and, to a less extent, to mature B cell lymphoma 2/10 samples (1 diffuse large B cell lymphoma (DLBCL) and 1 T-cell/histiocyte rich B cell lymphoma) in which neoplastic cells could not be identified by MFC, leading to false negative results. Because STOT did not allow identification of HL cells, we finally re-evaluated the contribution of simultaneous staining of STOT and the HL tube in lymph node samples in which no tumor cells had been detected by STOT (reactive profile). Based on this approach, diagnosis of HL was reached in 5/8 samples leading to an (overall) degree of agreement of 96% (143/149 samples) between the (STOT plus the HL tube) MFC screening approach and the WHO/ICCC-3 criteria for diagnosis of pediatric hematopoietic and non-hematopoietic tumors vs. reactive conditions and/or tumor-free patient samples (Table 3). Overall, these results translated into a diagnostic sensitivity of 93% and specificity of 100% (negative and positive predictive values of 91.3% and 100%, respectively) for STOT, once combined with the HL tube in lymph node samples showing no tumor infiltration by STOT.

## 3. Discussion

Fast and accurate diagnosis of pediatric cancer is key for (early) adequate treatment intervention and improved patient outcome [2,3]. At present, diagnosis of leukemia/lymphoma and other hematopoietic malignancies relies to a large extent on MFC immunophenotyping [11,24], in addition to cytomorphology/histopathology and molecular data [23]. In contrast, final diagnosis of non-hematopoietic (solid) tumors in children and adolescents is based on histopathological examination of tissue samples followed by IHC stainings for relatively broad panels of markers, together with further molecular investigations in specific diagnostic tumor subtypes [30]. Such approach used for the diagnostic screening of solid tumors in general is laborious and time consuming, leading to delayed final diagnosis in a substantial fraction of patients. [2,5,31,32] Altogether, this indicates that although MFC is a cornerstone in the diagnosis of leukemia/lymphoma in children, and it has been shown to be useful also for pediatric solid tumors [22], MFC is currently not part of the diagnostic workup of pediatric solid tumors, and no MFC standardized protocol has been proposed so far in this regard [9,20,33,34,35]. Here we designed a single 8-color/12-marker antibody combination (STOT) for diagnostic orientation and classification of pediatric solid tumors; subsequently, STOT was prospectively validated in a large cohort of samples from a wide variety of different tissues and WHO/ICCC-3 diagnostic categories of pediatric cancer. Overall, STOT showed a high diagnostic accuracy, except for a small group of lymph node samples infiltrated by HL. In addition, STOT also provided accurate orientation towards the diagnostic subtype of both the non-hematopoietic and hematopoietic tumor cells identified, as discussed below in more detail.

A major concern at the moment of starting this study was related to preservation of cell viability for MFC and its potential impact on diagnostic accuracy in the setting of a multicentric study requiring sample transportation to a central laboratory in a large city such as Rio de Janeiro (Brazil). In this study, we demonstrated that cell viability remained within an acceptable range in the great majority of samples, although higher cell viability rates were observed in BM, blood, and other body fluid samples compared with tumor masses. Such results are in line with previous studies, which have shown that mechanical disaggregation maintains antigen expression and an acceptable number of viable cells for subsequent MFC analysis [36,37]. Surprisingly or not, correct diagnosis was even possible in the great majority (but not all) of samples with significantly reduced cell viability rates. In this regard, time between tissue collection and processing emerged as the main factor affecting specimen viability (data not shown), which is potentially due to an increased tissue ischemia and cell death when prolonged periods of time between sample collection and data acquisition in the flow cytometer occur [38,39,40]. Further studies are necessary to design, evaluate, and establish protocols that might contribute to improve preservation of cell integrity through, e.g., immediate addition of specific stabilization/preservation solutions, as adopted here for cerebrospinal fluid samples [41].

Once the feasibility of using fresh tissue samples for MFC had been demonstrated, our major goal focused on the design of a single antibody combination for diagnostic orientation and classification of solid tumors (i.e., STOT). Overall, the design of STOT relied on a total of 42 different markers selected from a larger set of markers reported in the literature, which were tested in a large series of pediatric samples suspicious of tumor infiltration. During the marker evaluation phases, a first group of backbone markers for identification of both non-hematopoietic and hematopoietic tumor cells vs. normal tissue cells was selected, based on multivariate analysis (e.g., PCA and CA). Subsequently, a second group of markers were chosen for further subclassification of (B vs. T vs. myeloid) leukemia/lymphoma and non-hematopoietic benign and malignant tumors (NBL vs. EES vs. RMS vs. WT vs. other tumors). This resulted in a combination of 10 markers consisting of CD45, CD56, _cy_CD3, _sm_CD3, and CD19 supplemented with CD99, _nu_myogenin, CD271, EpCAM, and GD2, which were merged into a single 8-color/10-antibody combination. Of note, this antibody combination allowed for an aberrant identification of non-neoplastic stromal cells and immune resident and infiltrating cells, in addition to non-hematopoietic and hematopoietic neoplastic cells. For further subsetting of T-cells and specific identification of monocytes and dendritic cells, the anti-CD4 and anti-CD8 antibodies were also added to (the first and the final versions) of STOT, prior to its validation.

Pediatric solid tumors can be broadly classified into hematopoietic and non-hematopoietic solid tumors. Therefore, during validation of STOT, the first goal was to determine its ability to discriminate between hematopoietic and non-hematopoietic tumor cells and distinguish them from residual immune and (other tissue) stromal cells, in order to guide subsequent tumor diagnosis and classification. Fully in line with previous observations [15,22,23,24,26,42,43], CD45 and CD56, together with CD19 and both cell surface membrane and cytoplasmic CD3, enabled identification of tumor infiltrating leucocytes, including the major subsets of T, B, and NK cells and coexisting hematopoietic and/or non-hematopoietic tumor cells, in virtually all tumor infiltrated samples by histopathology/IHC, except for lymph node specimens from HL patients. These results are in line with those previously described by Barrena et al. [44] in adult (lymph node) samples infiltrated by lymphoid tumors, which also failed to identify HL cells with a prototype of the Lymphoclonal^TM^ reagent used in phase 1 of this study. Importantly, no reactive/inflammatory (tumor free) samples were misdiagnosed as cancer [22], neither during the design phase nor during validation of STOT. Because of all the above results, only minor changes were required (in the position of the CD3 markers) to transition from the β version to the final STOT antibody combination, apart from the addition of a second antibody combination specific to screen for HL cells in lymph node specimens showing no tumor infiltration with STOT. This additional HL-oriented antibody combination identified tumor cells in the majority (but not all) HL infiltrated samples, including all those samples in which >1 million cells had been evaluated. Further studies are thereby required to ensure whether the diagnostic efficiency of MFC in HL cases depends or not on the sensitivity of the assay and, consequently, on the number of lymph node cells measured as previously suggested [45,46].

Despite CD45 was critical for the differential diagnosis between hematopoietic and non-hematopoietic tumors, misdiagnosis of CD45^-/+lo^ hematopoietic neoplasms as non-hematopoietic tumor cells might occur. However, co-expression on CD45^−^ hematopoietic tumor cells of either B cell markers (i.e., CD19) or T-cell-associated proteins (e.g., _cy_CD3, _sm_CD3), usually in the absence of CD56 [47,48], were critical for correct hematopoietic vs. non-hematopoietic lineage assignment in these patients. In addition, the above markers also allowed diagnostic orientation toward a B, T, or non-B–non-T myeloid-lineage hematopoietic neoplasm. In contrast, their value for the differential diagnosis of SRCT and other less frequent non-hematopoietic tumors, as well as for HL, was rather limited. Because of this, a minor set of five additional markers (CD99, GD2, CD271, EpCAM, and _nu_myogenin) was selected from all tumor-associated proteins investigated, for the differential diagnosis between the four major subgroups of extracranial SRCT vs. other pediatric tumors.

The great majority (≈ 80%) of all pediatric solid tumors are classified as small round cell tumors (SRCT), which consist of highly proliferating malignant tumors with overlapping and undistinguishable morphologic features [49]. Because of this, further IHC stainings for a broad antibody specificities are typically required for the differential diagnosis between the distinct subtypes of SRCT [5]. Positivity for classical IHC markers, such as CD99 and both _nu_myogenin and _nu_MyoD1 [2,5,50], have long been associated with Ewing sarcoma and rhabdomyosarcoma, respectively. However, on their own, these markers still remain limited for accurate identification of these two diagnostic subtypes of pediatric cancer [2,5,51]. In turn, neuron-specific enolase (NSE), HISL19, and dopamine beta-hydroxylase have emerged as key markers for the diagnosis of neuroblastic tumors by IHC [2,5,51]. In parallel, MFC analysis of bone marrow metastatic neuroblastic tumors has highlighted the potential diagnostic utility of markers such as GD2 and CD81, together with CD9 and CD90 (apart from CD45 and CD56), in the diagnostic workup of these tumors [13,15,19,22,52]. Based on multivariate analysis [53], here we identified GD2, CD271, _nu_myogenin and/or _nu_MyoD1, CD99, and EpCAM to be the minimum set of markers required for the differential diagnosis of NBL (CD45^−^ CD56^++^ GD2^++^ CD271^−/+^ _nu_MyoD1^−^ _nu_myogenin^−^ CD99^−^ EpCAM^−^), EES (CD45^−^ CD56^++^ GD2^−/+^ CD271^+^ _nu_MyoD1^−^ _nu_myogenin^−^ CD99^+^ EpCAM^-^), RMS (CD45^−^ CD56^++^ GD2^−^ CD271^++^ _nu_MyoD1^++^ _nu_myogenin^++^ CD99^−/+^ EpCAM^−^), and WT (CD45^−^ CD56^++^ GD2^−^ CD271^+^ _nu_MyoD1^−^ _nu_myogenin^−^ CD99^−^ EpCAM^+^) vs. other pediatric tumors (e.g., CD45^+^ CD19^+^ CD56^−^ GD2^−^ CD271^−^_nu_MyoD1^−^ _nu_myogenin^−^ CD99^−^ EpCAM^−^ B-cell leukemia/lymphoma and CD45^−^ CD56^−^ GD2^−^ CD271^−^ _nu_MyoD1^−^ _nu_myogenin^−^ CD99^−^ EpCAM^++^ carcinomas). Based on these results, EpCAM, GD2, CD99, _nu_myogenin, and CD271 were included in STOT, in addition to CD45, CD56, and the other B- (e.g., CD19) and T-cell- (e.g., _sm_CD3, _cy_CD3, CD4, CD8) associated markers.

Prospective validation of STOT confirmed a high degree of accuracy of the new antibody combination for fast and accurate identification of both hematopoietic and non-hematopoietic tumor cells, as well as for orientation toward the most frequent subtypes of B-, T-, and myeloid malignancies vs. NBL, EES, WT, RMS, and other non-hematopoietic tumors. Furthermore, neoplastic cells from pediatric carcinomas could also be clearly distinguished from other hematopoietic and non-hematopoietic neoplastic cells based on their stronger expression of EpCAM compared with all other tumor subtypes. In contrast, despite a clear population of CD45^−^ tumor cells were systematically identified in GCT, STOT did not reveal a unique and reproducible immunophenotypic profile for GCT, due to the high heterogeneity observed in these later patients for the STOT markers. Altogether, these results point out the need for additional HL and GCT specific markers, such as CD30 and GPC3 [54], OCT3/4 [55], PLAP [56], for specific identification and classification of these two tumor subtypes, respectively. Overall, our results suggest that this standardized flow-cytometry approach may be of great help in the diagnostic workup of pediatric cancer. Despite MFC does not replace the conventional diagnostic procedures in pathology because it does not provide information on the structure and tissue location of tumor cells [9], it informs about patterns of antigen expression in single tumor cells, which help in guiding complementary immunohistochemical investigations. Further studies in close collaboration between pathologists and cytometrists are necessary to define the most robust, accurate, and efficient workflow for the integration of both methodologies. During validation of STOT, we were concerned about the ability of the STOT markers selected to discriminate between stromal cells and metastatic non-hematopoietic neoplastic cells, particularly in samples that contain a small fraction (<1%) of (non-hematopoietic) stromal cells, such as endothelial cells, and mesenchymal stromal cells (MSC) in BM [57]. In line with previous findings, MSC showed a CD45^−^ CD34^−^ CD81^++^ CD90^++^ CD271^++^ CD10^++^ phenotype, while endothelial cells were CD81^++^ CD10^−^ CD56^−^ CD271^+^ CD34^+^ [58,59,60]. These phenotypes contrasted with that of CD56^++^ GD2^++^ CD10^−^ CD34^−^ CD271^−/+^ neuroblastoma cells, which allows clear cut identification of neoplastic cells and their discrimination from normal stromal cells in BM, even when BM disseminated NBL cells were present at very low levels (data not shown). In this regard, it should be noted that minimal residual disease (MRD) assessment in blood and BM has been shown to provide prognostic information among pediatric patients diagnosed with non-hematopoietic solid tumors [17,57]. Thus, STOT may potentially became also useful for MRD monitoring and minimally invasive diagnostics in pediatric non-hematopoietic solid tumors, such as NBL. However, no clear distinction was possible between normal residual BM MSC and non-hematopoietic neoplastic cells in BM samples infiltrated by other types of cancer, such as rhabdomyosarcoma; this might lead to a lower sensitivity of detection of minimal numbers of CD45^−^ CD56^+^ non-hematopoietic tumor cells in BM in this subgroup of patients. Interestingly however, these findings point out a close phenotypic relationship (and overlap) between MSC and tumor cells in sarcoma patients, further studies being required to determine the biological and oncogenic basis for these similarities [61].

Finally, among those samples infiltrated by non-hematopoietic tumor cells, a major challenge was to distinguish between benign and malignant tumor cell types. Although a limited number of benign neoplasms was tested in this study, preliminary data suggest that CD45^−^ cells present in benign tumors display (aberrant) phenotypic features that more closely resemble those of normal non-hematopoietic cells from the same tissues (e.g., the cells from the tumor border in nephrectomy samples from patients with renal tumors) and that are completely different from those of malignant neoplastic cells. Thus, in line with previous studies, which showed that normal kidney cells express CD10, HLA-DR, and low EpCAM levels [62,63], these markers were also expressed here on all benign (but not malignant) kidney tumors. Likewise, endothelial cells precursor are known to display a CD34^++^ CD45^−^ phenotype together with positivity for CD10 and CD90 [64], similarly to the tumor cells from the few hemangioma tumors identified here, in line with what has been previously described for hemangioma-derived multipotent stem cells [65]. However, in contrast to normal endothelial cells, hemangioma cells lacked CD81 expression [66].

Despite the low numbers encountered, our study, also for the first time, provided data on the MFC immunophenotypic profiles of exceedingly rare neoplasms, such as a malignant nerve sheath tumor and a pseudopapillary tumor of the pancreas. Unexpectedly, GD2 expression was observed on the malignant nerve sheath tumor studied here. Further investigations in larger cohorts of patients diagnosed with these and other rare tumor subtypes are needed to better define their phenotypic profile and the potential diagnostic utility of STOT in such tumors.

In summary, here we designed and validated a single antibody combination for fast and accurate diagnostic screening, orientation, and classification of pediatric solid tumors, which might be used as a complementary tool to conventional histopathology for guiding diagnosis and classification of pediatric cancer. Further multicentric validation of STOT is now ongoing within the EuroFlow consortium and particular attention is being paid to the detection of HL cells and other rare non-hematopoietic tumor types.

## 4. Material and Methods

### 4.1. Patients and Samples

A total of 476 consecutive samples from 296 children and young adolescents—115 females and 181 males; median age of 7.3 years (y), ranging from 10 days to 19 y—suspicious of having a non-hematopoietic (solid) tumor (Table S3), were collected between May 2009 and February 2021 at 5 reference centers for Pediatric Oncology in Rio de Janeiro (Brazil)—Instituto de Puericultura e Pediatria Martagão Gesteira (IPPMG/UFRJ), Hospital Federal da Lagoa, Hospital Federal dos Servidores do Estado (HFSE), Hospital Estadual da Criança, and Rede D’or São Luiz—and included in this study. Most samples were studied at diagnosis (*n* = 423) and a smaller fraction at relapse (*n* = 22) or during follow-up (*n* = 31). Samples included 139 solid tumor mass specimens, 86 lymph node, 138 BM, and 58 peripheral blood (PB) samples, in addition to another 55 body fluid specimens (e.g., ascitic fluid, pleural effusion, urine, pericardial effusion, and cerebrospinal fluid) (Table S3 and Table 1). All samples were studied in parallel by MFC and histopathology/cytopathology and IHC, and whenever indicated, they were also investigated for specific cytogenetic/molecular alterations following in-house diagnostic algorithms and procedures. Prior to sample collection, written informed consent was given by each donor and/or his/her legal representative(s) according to the Declaration of Helsinki; the study was approved by the local ethics committees of the participating centers.

In all cases, final diagnosis and classification were established by two independent (experienced) pathologists based on the World Health Organization (WHO) and the International Classification of Childhood Cancer (ICCC-3) criteria [30,42]. The great majority of patients (216/296, 73%) had cancer, including metastatic disease in 89/216 (30%) cases, and 8 (3%) children were diagnosed with benign tumors (hemangioma, 2 cases; hemangiopericytoma, 1; neurofibroma, 1; cystic nephroma, 1; mesoblastic nephroma, 2; and benign cutaneous fibrous histiocytoma, 1 patient). The remaining 72/296 patients (24%) had inflammatory/reactive disease conditions. Among the 216 cancer patients, 111 (51%) were diagnosed with a hematologic malignancy, including BCP-ALL in 15 patients, T-LL/ALL in 23, AML in 4, and other lymphomas/reticuloendothelial neoplasms in 69 (23%) cases—BL, 26; HL, 24; ALCL, 6; DLBCL, 7; histiocytic disorders, 4; EBV-related T cell lymphoma, 1; and T-cell/histiocytic rich B cell lymphoma, 1 case. Among children with cancer, 105/216 (49%) patients had non-hematopoietic (malignant) solid tumors consisting of 45 (21%) NBL and other peripheral nervous cell tumors (41 NBL, 2 ganglioneuroblastomas, 1 ganglioneuroma, and 1 pheochromocytoma), 17 (7.8%) renal tumors (15 nephroblastoma/WT, 1 renal cell carcinoma, and 1 clear cell sarcoma), 5 (2.3%) malignant bone tumors (4 osteosarcomas and 1 chondrosarcoma), 16 (7.4%) soft tissue and other extraosseous sarcomas (8 RMS, 7 EES, and 1 malignant peripheral nerve sheath tumor), 10 (4.6%) GCT (6 immature teratomas, 3 yolk sac tumors and 1 dysgerminoma), 6 (2.7%) malignant epithelial neoplasms (3 adrenocortical carcinomas and 3 nasopharyngeal carcinomas), 2 (0.9%) hepatoblastomas, 1 (0.4%) medulloblastoma, 1 (0.4%) pseudopapillary pancreatic solid tumor (or Frantz tumor), and 2 (0.9%) unspecified malignant tumors (Table S3). Further data on the type of sample evaluated and the number of tumor infiltrated samples per WHO/ICCC-3 diagnostic tumor subtype are shown in detail in Table 1.

Histopathological examination of tissue samples was performed on hematoxylin and eosin (H&E)-stained (3 µm) tissue sections. Whenever tumor infiltration was suspected and/or detected, tissue sections were further stained via conventional IHC using a panel of markers adapted to the suspicious tumor subtypes based on the clinical data available and the histopathological and morphological features of tumor cells [2,5]. For liquid samples (e.g., blood, BM aspirates, urine, and other body fluids), conventional cytology plus cytochemistry were routinely performed.

### 4.2. Sample Preparation for Flow Cytometry

Once collected, samples were immersed in phosphate buffered saline (PBS; pH = 7.4), placed at 4 °C and transported to IPPMG/UFRJ for central MFC analysis. Briefly, solid tissue specimens free from fat and necrotic tissue (mean weight: 270 mg; range: < 4–5000 mg) were directly examined by an experienced pathologist and divided into two contiguous blocks. One block was fixed in formalin, embedded in paraffin, and processed for routine histopathology, while the second block was immersed in cold (4 °C) PBS and sent to the MFC laboratory, where it was processed within the first 6 h after collection. Prior to MFC sample preparation, each specimen aliquot was weighed, its physical description recorded, and divided into two fragments: one was immediately placed in a methanol/acetic acid solution (3/1 vol/vol) and stored at −20 °C for further interphase fluorescence in situ hybridization (FISH) and other genetic studies, while the other fragment was mechanically disaggregated and immediately processed for MFC, as previously described [22].

Body fluids (*n* = 55) were collected in either an EDTA tube (*n* = 53; 32 pleural effusions, 9 ascitic fluid, 8 pericardial effusions, and 4 urine samples) or, in the case of 2 cerebrospinal fluid samples (CSF), in a tube containing 5–10% volume of TransFix (Caltag Medsystems, Towcester, UK). Then, tubes containing the samples were placed on melting ice, centrifuged (at 800 g for 10 min), resuspended in 5 mL of PBS containing 0.2% bovine serum albumin (PBS–BSA), washed twice (at 540 g for 5 min) in PBS–BSA, and resuspended in 100μL of PBS–BSA. Subsequently, these samples, as well as BM and PB samples, were (immediately) processed following the EuroFlow stain-and-then-lyse standard operating procedures (SOPs) for staining of surface markers only or cell surface membrane plus intracellular markers, as detailed in Supplementary Methods (SOPs available at www.EuroFlow.org) [67,68]. For increased sensitivity of detection of circulating/metastatic tumor cells (CTC/MTC) in PB and BM, an erythrocyte bulk-lysis step was performed before staining in a subset of 66/138 BM (48%) and 33/58 PB (57%) specimens, strictly following the EuroFlow bulk-lysis SOP [67,68].

### 4.3. Antibody Combinations

For the design and validation of STOT, four different panels of antibody combinations were tested in sequential cycles of design–test–redesign on 104, 197, 140, and 35 samples, respectively (Figure 1 and Table 2). Briefly, a panel consisting of 8 antibody combinations of between 4 and 8 markers/tube, for a total of 42 distinct fluorochrome-conjugated and unconjugated mAbs was first designed based on our previous results [22] and tested. This panel consisted of two screening tubes (EuroFlow ALOT and Lymphoclonal^TM^) followed by a 6-tube panel aimed at further subclassification of non-hematopoietic tumors (Figure 1, Table 2 and Table S1). Evaluation of this first antibody panel focused on the identification of non-redundant markers for discrimination between normal, reactive, leukemia/lymphoma, and non-hematopoietic tumor cells, after it had been tested on a first set of 104 (consecutive) samples (Figure 1 and Figure 2).

In a second step, ALOT and LST (instead of ALOT and Lymphoclonal^TM^) were used, either alone or together with a third HL-oriented tube in the case of lymph node samples suspicious of an underlying reactive/inflammatory disease vs. HL, to stain 197 (additional) samples (Figure 1 and Table 2). This third HL monoclonal antibody combination was specifically devoted to the differential diagnosis between reactive lymphocytes and both HL and ALCL based on the CD20, CD45, CD15, CD30, CD5, CD56, CD123, and _sm_CD3 markers (Table 2), and it was systematically studied in lymph node samples from study phase 2 onward (Figure 1). In case of pediatric hematopoietic and non-hematopoietic tumors, samples were subsequently stained with ≥1 of the EuroFlow BCP-ALL, T-ALL, and/or AML/MDS classification panels or the solid tumor panel used in the first step of this study, respectively (Table S4).

Based on the results obtained with the above panel, a first version of the EuroFlow STOT tube (Table 2) was designed (step 3A) for identification, in a single tube, of the major populations of leukocytes coexisting in a sample with either hematopoietic and/or non-hematopoietic tumor cells, and further orientation toward the major diagnostic subtypes of hematopoietic tumors and SRCT. At this stage, STOT was evaluated in 140 additional samples (Figure 1). Pre-selection of fluorochrome-conjugated monoclonal antibody reagents for STOT was based on their mean fluorescent intensity (MFI), stain index (SI), and spectral overlap into other fluorescence channels, as previously described [69]. Evaluation of this first prototype version of STOT (β version) confirmed optimal performance of most reagents and the overall antibody combination, with the need for finetuning of only CD3 reagent positions. Thus, the position of _cy_CD3 (previously conjugated with the same fluorochrome as CD19) in STOT was inverted with that of _sm_CD3 (previously conjugated with the same fluorochrome as CD271) to improve the differential diagnosis between B and T cell neoplasias. The resulting final version of STOT was tested on another 35 samples (step 3B). Thus, the final configuration of STOT consisted of the following markers: CD99-CD8/_nu_myogenin/CD4-EpCAM/CD56/GD2/_sm_CD3-CD19/_cy_CD3-CD271/CD45 (Figure 1 and Table 2).

### 4.4. Data Acquisition and Analysis

MFC data acquisition was performed on a FACS Canto II flow cytometer (BD) using the FACSDiva software (version 8, BD, San José, CA, USA). For BM and PB samples, as well as for lymph node samples suspicious of HL, ≥ 5 × 10^6^ cellular events per tube were measured, while for all other samples, >10^4^ cells per tube were evaluated (median: 5 × 10^5^ cells/tube; range: 10^4^–10^7^ cells/tube) unless sufficient cells were not available. For data analysis, the Infinicyt^TM^ software (Cytognos SL, Salamanca, Spain) was employed. Gating of non-hematopoietic tumor cells and normal residual cells, as well as the distinct subsets of leukocytes, was performed by an expert using Boolean gating strategies, after cell debris and doublets had been excluded, as previously described in detail [8] and illustrated in Figure 2. Antigen expression was used to identify the different types of cells present in the sample, and each cell population identified was classified as being negative (−), dim positive (lo), intermediate positive (+), or strongly positive (++), using arbitrary relative linear mean fluorescence intensity (MFI) values (vs. baseline autofluorescence levels found in the control tube) of 10^0^–10^2^, 10^2^–10^3^, 10^3^–10^4^, > 10^4^, respectively; in turn, antigen expression for a given marker was described as heterogeneous when variable expression levels were detected for that marker within the tumor cell population.

In order to identify those markers that provided independent (i.e., non-redundant) diagnostic information at each of the above phases, principal component analysis (PCA) and canonical analysis (CA) of merged MFC data files from those patient samples stained with the same antibody panels was performed, based on the automatic population separator (APS) and canonical analysis (CA) tools of Infinicyt^TM^ (Figure 2 and Figure S1). In parallel, a second merged MFC data file was also used at each of the above steps, which included MFC data of specifically gated non-hematopoietic tumor cells from all FCS data files in which they had been identified. This second merged MFC data file was uploaded in Infinicyt^TM^ as a database for subsequent automated classification of all non-hematopoietic tumor subtypes included in the database during the validation phase (phase 3) of the study (Figure 2).

### 4.5. Flow Cytometric Identification and Exclusion of Non-Viable Cells and Cell Debris

For reproducible identification of all nucleated cells and leukocytes present in individual samples, three protocols were initially evaluated in parallel in 5 samples: i) staining with propidium iodide (PI) (Sigma-Aldrich, St. Louis, MO, USA) plus CD45 (clone HI30, Exbio, Vestec, Czech Republic) [70]; ii) labeling with the DRAQ5^TM^ DNA dye (Biostatus, Cambridge, UK) plus CD45 (clone, HI30, Exbio, Vestec, Czech Republic) and CD56 (N901, Beckman Coulter, CA, USA) [71]; and, iii) a Boolean gating strategy for exclusion of dead cells and cell debris based on the forward (FSC) and sideward (SSC) light-scatter characteristics and the immunophenotypic pattern of CD45^+^ leucocytes and CD45^−^ non-hematopoietic cells. For these experiments, ≥2 × 10^4^ cells/tube were measured in a FACSCanto II flow cytometer, as described above.

### 4.6. Statistical Methods

For all continuous variables, median and mean values, and their standard deviation (SD), as well as overall and interquartile ranges (IQR) and the 5th and 95th percentiles, were calculated. For categorical variables, frequencies were used. To evaluate the sensitivity, specificity, accuracy, positive predictive value (PPV), and negative predictive value (NPV) of MFC, results were compared with the final WHO/ICCC-3 diagnoses (gold standard). To establish the statistical significance of differences observed between groups, the Mann–Whitney U test and the χ^2^ test were used for continuous and categorical variables, respectively. For all statistical analyses, the IBM-SPSS software program (version 18.0, IBM, Chicago, IL, USA) was used. *p*-values < 0.05 were considered to be associated with statistical significance.

## 5. Conclusions

Simultaneous assessment of CD45 and CD56 together with (surface membrane and cytoplasmic) CD3 and CD19, as well as _nu_myogenin, EpCAM, CD271, and GD2, provides fast and highly accurate diagnostic orientation in children suspicious of having cancer. In addition, STOT provides information about the cellular composition of the tumor microenvironment, particularly when CD4 and CD8 are added to the CD3, CD19, CD45, CD56, and CD271 markers.

## Figures and Tables

**Table 1:** Number of infiltrated samples with percentage of tumor cells and viable cells after excluding dead cells and cell debris, and distribution by sites analyzed, grouped according to the final (WHO/ICCC-3) diagnosis (*n* = 476 samples/296 patients).

WHO/ICCC-3 Diagnosis (*n* = 476)	Nº of Infiltrated/ Total samples (%)	% Tumor Cells *	% Viable Cells *	Type of Sample				
				Tumor Mass (%)	BM (%)	PB (%)	Lymph Node (%)	Other Fluid (%)
Hematological malignancies (*n* = 194)	118/194 (61%)			34/139 (24%)	76/138 (55%)	18/58 (31%)	34/86 (39%)	32/55 (58%)
Leukemias/lymphoblastic lymphomas (*n* = 82)	56/82 (68%)	57 (0.5–99)	66 (11–90)	20/34 (59%)	28/76 (37%)	10/18 (55%)	7/34 (20%)	17/32 (53%)
Acute myeloid leukemia (*n* = 8)	8/8(100%)	36 (0.7–99)	67 (48–88)	2/20 (10%)	4/28 (14%)	2/10 (20%)	-	-
B-cell precursor lymphoblastic leukemia/lymphoma (*n* = 22)	16/22(72%)	51 (0.5–98)	66 (11–90)	5 ^§^/20 (25%)	10/28 (36%)	1/10 (10%)	3/7 (43%)	3/17 (18%)
T-cell lymphoblastic lymphoma/leukemia (*n* = 52)	31/52 (59%)	66 (0.9–99)	65 (11–90)	13/20 (65%)	14/28 (50%)	7/10 (70%)	4/7 (37%)	14 ^ɸ^/17 (82%)
Lymphomas and reticuloendothelial neoplasms (*n* = 112)	62/112 (55%)	43 (0.003–97)	68 (1–97)	14/34 (41%)	48/76 (63%)	8/18 (44%)	27/34 (80%)	15/32 (47%)
Diffuse large B cell lymphoma (*n* = 11)	7/11 (64%)	41 (1.5–93)	64 (20–89)	1/14 (7%)	4/48 (9%)	-	4/27 (15%)	2/15 (13%)
Anaplastic large cell lymphoma (*n* = 10)	8/10 (80%)	19 (0.1–49)	76 (34–90)	1/14 (7%)	4/48 (9%)	1/8 (12%)	1/27 (4%)	3/15 (20%)
Burkitt lymphoma (*n* = 40)	20/40 (50%)	66 (0.09–96)	67 (1–93)	5/14 (36%)	20/48 (41%)	3/8 (40%)	6/27 (21%)	6 ^ɸ^/15 (40%)
EBV-related lymphoma (*n* = 3)	3/3(100%)	87 (69–97)	77 (72–82)	1/14 (7%)	1/48 (2%)	1/8 (12%)	-	-
T-cell/histiocyte rich B cell lymphoma (*n* = 3)	1/3 (33%)	0 ^#^	35 (2.5–75)	-	-	1/8 (12%)	1/27 (4%)	1/15 (7%)
Hodgkin lymphoma (*n* = 39)	19/39 (49%)	0.6 (0.003–3)	69 (12–97)	5/14 (36%)	15/48 (31%)	2/8 (24%)	14/27 (52%)	3/15 (20%)
Histiocytic sarcoma (*n* = 2)	1/2 (50%)	27	65 (47–83)	-	2/48 (4%)	-	-	-
Langerhans cell histiocytosis (*n* = 4)	3/4 (75%)	0.4 (0.01–27)	68 (38–87)	1/14 (7%)	2/48 (4%)	-	1/27 (4%)	-
Non-hematopoietic pediatric cancer (*n* = 178)	110/178 (62%)			82/139 (59%)	51/138 (37%)	31/58 (53%)	3/86 (4%)	11/55 (20%)
Neuroblastoma and other peripheral nervous cell tumors (*n* = 79)	44/79 (55%)	32 (0.008–97)	62 (2–97)	24/82 (29%)	40/51 (78%)	13/31 (42%)	2/3 (67%)	0/11 (0%)
Neuroblastoma (*n* = 71)	38/71 (53%)	30 (0.008–97)	65 (2–97)	20/24 (83%)	38/40 (95%)	12/13 (92%)	1/2 (50%)	-
Ganglioneuroblastoma (*n* = 4)	4/4 (100%)	46.6 (24–93)	25.2 (7–55)	2/24 (8%)	1/40 (2.5%)	-	1/2 (50%)	-
Ganglioneuroma (*n* = 2)	1/2 (50%)	86	49 (21–78)	1/24 (4%)	1/40 (2.5%)	-	-	-
Pheochromocytoma (*n* = 2)	1/2 (50%)	16	50 (10–90)	1/24 (4%)	-	1/13 (8%)	-	-
Kidney tumors (*n* = 22)	17/22 (77%)	68 (1.5–99)	38 (1.3–89)	18/82 (22%)	0/51 (0%)	3/31 (9%)	0/3 (0%)	1/11 (9%)
Nephroblastoma / Wilms Tumor (*n* = 19)	15/19 (79%)	65 (1.5–99)	37 (1.3–89)	16^&^/18 (89%)	-	2/3 (67%)	-	1/1(100%)
Clear cell sarcoma (*n* = 2)	1/2(50%)	51	56 (46–67)	1/18 (5.5%)	-	1/3 (33%)	-	-
Renal cell carcinoma (*n* = 1)	1/1(100%)	76	2	1/18 (5.5%)	-	-	-	-
Liver tumors (*n* = 2)	2/2 (100%)	62	4.4 (2–7)	2/82 (2.4%)	0/51 (0%)	0/31 (0%)	0/3 (0%)	0/11 (0%)
Hepatoblastoma (*n* = 2)	2/2 (100%)	62	4.4 (2–7)	2/2 (100%)	-	-	-	-
Malignant bone tumors (*n* = 8)	4/8 (50%)	32 (0.8–88)	40 (1–67)	4/82 (5%)	0/51 (0%)	2/31 (6%)	0/3 (0%)	2/11 (18%)
Osteosarcoma (*n* = 7)	3/7 (28%)	4 (0.8–7)	39 (1–67)	3/4 (75%)	-	2/2 (100%)	-	2/2 (100%)
Chondrosarcoma (*n* = 1)	1/1 (100%)	88	50	1/4 (25%)	-	-	-	-
Soft tissue and other extraosseous sarcomas (*n* = 36)	20/36 (55%)	50 (0.3–100)	53 (2.4–90)	12/82 (14%)	9/51 (17%)	8/31 (26%)	1/3 (33%)	6/11 (54%)
Rhabdomyosarcoma (*n* = 17)	13/17 (76%)	45 (0.3–100)	45 (2.4–82)	5/12 (41%)	4/9 (45%)	2/8 25%)	-	6/6 (100%)
Extraosseous Ewing sarcoma (*n* = 17)	6/17 (35%)	72 (52–92)	62 (9–90)	6/12 (50%)	5/9 (55%)	5/8 (63%)	1/1 (100%)	-
Malignant nerve sheath tumor (*n* = 2)	1/2 (50%)	14	52 (23–81)	1/12 (9%)	-	1/8 (12%)	-	-
Germ cell tumors, trophoblastic tumors, and neoplasms gonads (*n* = 16)	13/16 (81%)	32 (1−96)	25 (6–74)	12/82 (14%)	0/51 (0%)	2/31 (6%)	0/3 (0%)	2/11 (18%)
Germ cell tumor (*n* = 16)	13/16 (81%)	32 (1–96)	25 (6–74)	12/12 (100%)	-	2/2 (100%)	-	2/2 (100%)
Malignant epithelial neoplasms (*n* = 9)	7/9 (77%)	66 (4–87)	39 (12–71)	7/82 (8.5%)	0/51 (0%)	2/31 (6%)	0/3 (0%)	0/11 (0%)
Nasopharyngeal carcinoma (*n* = 4)	3/4 (75%)	36 (4–69)	54 (20–64)	3/7 (43%)	-	1/2 (50%)	-	-
Adrenal carcinoma (*n* = 5)	4/5 (80%)	80 (70–87)	70	4/7 (57%)	-	1/2 (50%)	-	-
CNS miscellaneous intracranial and intraspinal neoplasms (*n* = 3)	0/3 (0%)	0	80 (71–88)	0/82 (0%)	2/51 (4%)	1/31 (3%)	0/3 (0%)	0/11 (0%)
Medulloblastoma (*n* = 3)	0/3 (0%)	0	80 (71–88)	-	2/2 (100%)	1/1 (100%)	-	-
Other rare malignant neoplasms (*n* = 3)	3/3 (100%)	59 (11–96)	16 (3–40)	3/82 (3.6%)	0/51 (0%)	0/31 (0%)	0/3 (0%)	0/11 (0%)
Undifferentiated malignant neoplasm (*n* = 2)	2/2 (100%)	40 (11–70)	3.5 (3.5–4)	2/3 (66%)	-	-	-	-
Pseudopapillary pancreatic solid tumor (*n* = 1)	1/1 (100%)	96	40	1/3 (34%)	-	-	-	-
BENIGN NEOPLASMS (*n* = 9)	8/9 (89%)	NA	31 (4–63)	8/139 (6%)	0/138 (0%)	1/58 (2%)	0/86 (0%)	0/55 (0%)
Mesoblastic nephroma (*n* = 2)	2/2 (100%)	NA	28 (11–48)	2/8 (25%)	-	-	-	-
Cystic nephroma (*n* = 1)	1/1 (100%)	NA	10	1/8 (12.5%)	-	-	-	-
Hemangioma (*n* = 3)	2/3 (66%)	NA	24 (4–63)	2/8 (25%)	-	1/1(100%)	-	-
Hemangiopericytoma (*n* = 1)	1/1 (100%)	NA	43	1/8 (12.5%)	-	-	-	-
Neurofibroma (*n* = 1)	1/1 (100%)	NA	16	1/8 (12.5%)	-	-	-	-
Benign cutaneous fibrohistiocytic tumor (*n* = 1)	1/1 (100%)	NA	81	1/8 (12.5%)	-	-	-	-
No neoplastic disease (*n* = 95)	0/95 (0%)	NA	60 (0.7–93)	15/139 (11%)	11/138 (8%)	8/58 (14%)	49/86 (57%)	12/55 (22%)
Total	236/476 (49%)	-	59 (0.7–98)	139/476 (29%)	138/476 (29%)	58/476 (12%)	86/476 (18%)	55/476 (12%)

^#^ No diagnosis was obtained by MFC. * Results expressed as number of samples/total samples (percentages) or as median (range) percentage of cells ^&^ 15 Wilms tumor masses and 1 non-infiltrated tumor border; ^§^ 4 infiltrated tumor masses and 1 non-infiltrated contralateral testis; ^ɸ^ 2 cerebrospinal fluid samples; WHO/ICCC-3: World Health Organization/International Classification of Childhood Cancer 3; BM: Bone marrow; PB: peripheral blood; NA: not applicable.

**Table 2:** Antibody combinations evaluated along the different cycles of ‘design–test–redesign’ until the final version of solid tumor orientation tube (STOT) was built for the diagnostic screening and classification of pediatric non-hematopoietic tumors.

Study Phase	Antibody Panels	Fluorochrome-Conjugated Reagents							
		PB/BV421	PO	FITC	PE	PerCPCy5.5	PE–Cy7	APC	APCH7
	Lymphoclonal^TM^	CD20 ^§^	CD45 ^§^	CD8 + Igλ ^§^	CD56 + Igκ ^§^	CD4 + CD19 ^§^	CD56 ^§^	CD3 ^§^	–
PHASE 1	ALOT	cyCD3 ᵠ	CD45 ᵠ	cyMPO ᵠ	cyCD79a ᵠ	CD34 ᵠ	CD19 ᵠ	CD7 ᵠ	smCD3 ᵠ
	Solid tumor panel ^#^	*–*	CD45	CD57	CD90	CD34	CD56	Epcam	–
		*–*	CD45	CD99	CD81	CD9	CD56	CD117	–
		*–*	CD45	CD58	CD38	–	CD56	CD10	–
		*–*	CD45	–	CD271	–	CD56	–	–
		*–*	–	nuMyoD	nuMyogenin	GD2	cyDesmin	–	–
	LST	CD20 + CD4 ᶱ	CD45 ᶱ	CD8 + Igλ ᶱ	CD56 + Igκ ᶱ	CD5 ᶱ	CD19 + TCRγδ ᶱ	CD3 ᶱ	CD38 ᶱ
PHASE 2	ALOT	cyCD3 ᵠ	CD45 ᵠ	cyMPO ᵠ	cyCD79a ᵠ	CD34 ᵠ	CD19 ᵠ	CD7 ᵠ	smCD3 ᵠ
	HL/ALCL	CD20 *	CD45 *	CD15 *	CD30 *	CD5 *	CD56 *	CD123 *	smCD3 *
	Solid tumor panel ^#^	*–*	CD45	CD57	CD271	–	CD56	CD117	–
		*–*	CD45	nuMyoD	nuMyogenin	–	CD56	CD10	CD38
		*–*	CD45	CD44	CD105	CD9	CD56	CD71	CD81
		*–*	CD45	GD2	CD90	–	CD56	–	–
	Markers	smCD3 + CD271(BV421)	CD45	CD99 + CD8	nuMyogenin	Epcam + CD4	CD56	GD2	cyCD3 + CD19
PHASE 3A	STOT (Βeta version) Clone	UCHT1+ C40-1457	HI30	Tü12/UCH-T4	F5D	SK3/EBA-1	N901	14.G2a	SK7 + SJ25C1
	Source	BD	Exbio	BD/Cytognos	BD	Cytognos/BD	Beckman Coulter	BD	BD/
									Beckman Coulter
PHASE 3B	Markers	cyCD3 + CD271(BV421)	CD45	CD99 + CD8	nuMyogenin	Epcam + CD4	CD56	GD2	smCD3 + CD19
	STOT (Final version) Clone	UCHT1+ C40-1457	HI30	3B2TA8/UCH-T4	F5D	SK3/EBA-1	N901	14.G2a	SK7 + SJ25C1
	Source	BD	Invitrogen	BD/Cytognos	BD	Cytognos/BD	Beckman Coulter	BD	BD/
									Beckman Coulter

^§^ Lymphoclonal^TM^-9 kit (Cytognos SL, Salamanca, Spain); ᵠ ALOT—acute leukemia orientation tube (EuroFlow Consortium); ^#^ in 11 samples, a reduced panel for classification of solid tumors was used; ᶱ LST—lymphocyte screening tube; HL—Hodgkin lymphoma tube; * combination tested for Hodgkin lymphoma as well as for anaplastic large cell (72 samples). In case of hematopoietic tumors, the EuroFlow BCP-ALL, T-ALL, and AML classification panels were used after the screening tubes. Abbreviations (in alphabetical order): APC—allophycocyanin; APCH7—allophycocyanin–hilite 7; BD—Becton/Dickinson Biosciences; BV—brilliant violet; cy—cytoplasmic; FITC—fluorescein isothiocyanate; PerCPCy5.5—peridinin–chlorophyll protein-complex cyanin 5.5; PE—phycoerythrin; PE–Cy7—PE–cyanin 7.

**Table 3:** Validation of STOT alone (**A**) or together with the HL tube (**B**) for the diagnostic screening, orientation, and classification of non-hematopoietic and hematopoietic (solid) tumors: comparison with the conventional WHO/ICCC-3 diagnosis.

**A. STOT: Diagnostic Concordance with WHO/ICCC-3 Diagnosis**		
Flow cytometry	WHO/ICCC-3 diagnosis	
	No tumor infiltration (*n* = 63) *	Tumor infiltration (*n* = 86) *
Disease free/reactive	63/63 (100%)	11/86 (12%)
Non-hematopoietic tumors	1/48 (2%)	47/48 (98%)
Benign	0/4 (0%)	4/4 (100%)
Malignant	1/44 (2%)	43/44 (98%)
NBL	0/13 (0%)	13/13 (100%)
EES	0/2 (0%)	2/2 (100%)
RMS	0/5 (0%)	5/5 (100%)
WT	0/7 (0%)	7/7 (100%)
Other	1/17 (6%) ^♣^	16/17 (94%)
Hematopoietic tumors	10/38 (3%)	28/38 (74%)
AML/MS	0/4 (0%)	4/4 (100%)
T-ALL/LL	0/13 (0%)	13/13 (100%)
BCP-ALL/LL	0/1 (0%)	1/1 (100%)
BL	0/6 (0%)	6/6 (100%)
DLBCL	1/3 (33%)	2/3 (66%)
T-cell/histiocyte rich B-cell lymphoma	1/1 (100%)	0/1 (0%)
Anaplastic large cell lymphoma	0/2 (0%)	2/2 (100%)
HL	8/8 (100%)	0 (0%)
**B. STOT Plus HL Tube: Diagnostic Concordance with WHO/ICCC-3 Diagnosis**		
Flow cytometry	WHO/ICCC-3 diagnosis	
	No tumor infiltration (*n* = 63)	Tumor infiltration (*n* = 86)
Non-hematopoietic tumors	1/48 (2%)	47/48 (98%)
Benign	0/4 (0%)	4/4 (100%)
Malignant	1/44 (2%)	43/44 (98%)
NBL	0/13 (0%)	13/13 (100%)
EES	0/2 (0%)	2/2 (100%)
RMS	0/5 (0%)	5/5 (100%)
WT	0/7 (0%)	7/7 (100%)
Other	1/17 (6%) ^♣^	16/17 (94%)
Hematopoietic tumors	5/38 (13%)	33/38 (87%)
AML/MS	0/4 (0%)	4/4 (100%)
T-ALL/LL	0/13 (0%)	13/13 (100%)
BCP-ALL/LL	0/1 (0%)	1/1 (100%)
BL	0/6 (0%)	6/6 (100%)
DLBCL	1/3 (33%)	2/3 (66%)
T-cell/histiocyte rich B-cell lymphoma	1/1 (100%)	0/1 (0%)
Anaplastic large cell lymphoma	0/2 (0%)	2/2 (100%)
HL	3/8 (37%)	5/8 (63%)

* Results expressed as number of samples/total samples (percentages). ^♣^ One osteosarcoma sample.

## Supplementary Materials

The following are available online at https://www.mdpi.com/article/10.3390/cancers13194945/s1: Figure S1: Schematic representation of the data file merge and calculation procedure used for construction of the database, Table S1: Monoclonal antibody reagents during the design, construction, and testing of the Solid Tumor Orientation Tube (STOT), Table S2: Pattern of antigen expression by tumor cells from different diagnostic categories of pediatric solid tumors, Table S3: Demographic and clinical features of pediatric patients (*n* = 296) and their samples (*n* = 476) analyzed in this study, Table S4: EuroFlow panels of antibody combinations for diagnostic classification of B-cell precursor and T-cell acute lymphoblastic leukemia/lymphoma, and acute myeloid leukemia following the acute leukemia orientation tube (ALOT).
