# Supplementary material for: Flow Cytometry Immunophenotyping for Diagnostic Orientation and Classification of Pediatric Cancer Based on the EuroFlow Solid Tumor Orientation Tube (STOT)
Source: Cancers (Basel). 2021 Sep 30;13(19):4945. doi: 10.3390/cancers13194945 (PMC8508207; doi:10.3390/cancers13194945)
Supplement: Supplementary file 1 [file cancers-13-04945-s001.zip › cancers-1358347-SI.pdf]

cancers

MDPI

Article

Supplementary Metails: Flow Cytometry Immunophenotyping for Diagnostic Orientation and Classification of Pediatric Cancer Based on the EuroFlow Solid Tumor Orientation Tube (STOT)

Cristiane de Sá Ferreira-Facio, Vitor Botafogo, Patrícia Ferrão, Maria Clara Canellas, Cristiane Milito, Sérgio Romano, Daiana Lopes, Lisandra Teixeira, Elen Oliveira, Enrico Bruno-Riscarolli, Fabiana Mello, Patrícia Siqueira, Patrícia Moura, Francisco Nicanor Macedo, Danielle Forny, Luíza Simião, Ana Luíza Pureza, Marcelo Gerardin Poirot Land, Carlos Eduardo Pedreira, Jacques J. M. van Dongen, lberto Orfao and Elaine Sobral Costa

1. Methods

1.1. Design of the Solid Tumor Orientation Tube (STOT)

For every flow cytometric assay performed, EuroFlow standard operating procedures (SOPs) for sample preparation and staining were used. Staining was performed in 100µL of sample, to which the appropriate volume of fluorochrome-conjugated antibody reagents directed against cell surface markers was added. Samples were incubated with monoclonal antibodies for 30 min at room temperature (RT). When only staining for cell surface markers was performed, 2 mL of FACS lysing solution -Becton/Dickinson Biosciences (BD), San Jose, CA- diluted 1:10 (vol/vol) in distilled water was subsequently added, followed by an incubation for another 10 min at RT, and a washing step, after which cells were resuspended in 500 µL of phosphate buffered saline containing 0.2% bovine serum albumin (PBS-BSA). In turn, when surface membrane plus cytoplasmic markers were simultaneously assessed, after surface membrane staining, the cell suspension was washed and incubated for 15 min at RT with solution A of the Fix & Perm Reagent Kit (Nordic-MUbio, Susteren, The Netherlands). Afterward, washed cells were incubated for another 15 min at RT with solution B of the Fix & Perm Reagent Kit and antibodies against the intracellular markers. Stained cells were washed once and resuspended in 500 µL of PBS-BSA. For both protocols, cells were acquired immediately after sample preparation. All EuroFlow SOPs are freely available in full at www.EuroFlow.org.

1.2. Inclusion Criteria for Flow Cytometry Data Files in the Database and Database Construction

The actual criteria used for the selection of appropriate flow cytometry datafiles for construction of the MFC database were as follows: a) > 1×10^4 neoplastic cellular events; b) percentage of cell debris/doublets < 50%; c) homogeneous acquisition vs. time; d) acquisition with the appropriate and specific fluorochrome compensation matrix in the flow cytometer; and e) correct staining, based on external and internal negative and positive controls. In general, samples were excluded when they fulfilled more than one deviation, but in case of critical deviations, only one deviation was required for their exclusion from the database.

For database construction, sequential data merge and calculation of data in the MFC data files corresponding to tumor infiltrated samples was performed, as previously described [35], using FSC, SSC, CD45 and CD56 as the common backbone markers/parameters. Thus, staining profile for tumor cells for all remaining markers stained only with a fraction of the antibody combinations, was subsequently calculated, as previously described [7]. Afterward, tumor cells in the merged data files were classified by a color code according to the specific different WHO diagnosis, for a total of 12 distinct diagnostic

groups of cells: neuroblastic tumors (neuroblastoma, ganglioneuroblastoma, ganglioneuroma), extraosseous Ewing sarcoma, rhabdomyosarcoma, Wilms tumor, undifferentiated malignant neoplasm, osteosarcoma, renal cell carcinoma, pheochromocytoma, adrenal carcinoma, germ cell tumor, nasopharyngeal carcinoma and chondrosarcoma.

The analyzed merged flow cytometry data file was uploaded in Infinicyt™ software (Cytognos SL, Salamanca, Spain). To evaluate the discriminatory power of different combinations of markers for the identification of the different WHO/ICCC-3 diagnostic categories, balanced supervised principal component analysis (PCA) was performed using the automatic population separator (APS) tool of Infinicyt™. Markers with the higher discriminatory ability in each of the three conditions evaluated were selected for inclusion in STOT, as illustrated in Figure 2.

Table S1. Monoclonal antibody reagents evaluated during the design, construction, and testing of the Solid Tumor Orientation Tube (STOT).

Antibody Reagents					
**Marker**	**Clone**	**Fluorochrome**	**Source**	**Catalogue number**	**Aim**
CD3	UCHT1	BV421	BD	562426	TLL
	UCHT1	PB	BD	558117	GIT
	SK7	APC-H7	BD	641397	
CD4	SK3	PERCP-cy5.5	BD	341654	TLL
	RPA-T4	PB	Cytognos	300521	Monocyte, T-cell
CD5	L17F12	PerCPcy5.5	BD	341089	TLL, T-cell
CD7	124-1D1	APC	eBioscience	17-0079-42	TLL, T-cell
CD8	UCHT-4	FITC	Cytognos	Cyt-8F8	TLL, T-cell
CD9	M-L13	PerCPcy5.5	BD	341649	NHST
CD10	HI10a	APC	BD	332777	TLL, GCT, Sarcoma
CD15	MMA	FITC	BD	332778	HD
CD19	J3-119	PE-Cy7	Beckman/Coulter	IM3628	B-cell, BLL, MBCL
	SJ25C1	APC-H7	BD	641395	
CD20	2H7	PB	Biolegend	302320	B-cell, BLL, MBCL
CD22	S-HCL-1	APC	BD	333145	B-cell, BLL, MBCL
CD24	ML5	APC-H7	BD	658331	B-cell, BLL, MBCL
CD27	O323	BV421	Biolegend	562513	B-cell, MBCL
CD30	BerH8	PE	BD	550041	ALCL, HD
CD34	8G12	PerCPcy5.5	BD	347203	BLL, TLL
CD38	HB-7	PE	BD	347687	B-cell, BLL, MBCL
	HB-7	APC-H7	BD	656646	
CD44	L178	FITC	BD	347943	NHST
CD45	HI30	PO	Exbio	P0684T100	Leu
CD56	N901(NKH-1)	PE-Cy7	Beckman/Coulter	A21692	NHST, NK cell, T-cell
CD57	HNK-1	FITC	BD	347393	NHST
CD58	1C3	FITC	BD	555920	NHST
CD71	M-A712	APC-H7	BD	655408	NHST
cyCD79a	HM57	PE	Dako	R7159	B-cell, BLL, MBCL
CD81	JS-81	PE	BD	555676	NHST, BLL
	JS-81	APC-H7	BD	656647	MBCL
CD90	5.00E + 10	PE	BD	555596	NHST
CD99	TÜ12	FITC	BD	555688	TLL, EWS
	TÜ12	PE	BD	555689	
CD105	TEA3/17.1.1	PE	Beckman/Coulter	PNA07414	NHST, EDC

CD117	104D2	APC	BD	341096	NHST
CD123	AC145	APC		130-113-322	DC, NHST, HD
CD271	C40-1457	PE	BD	557196	Sarcoma
	C40-1457	HV450	BD	562123	WT, NBL
	C40-1457	BV421	BD	562562	EWS, MSC
CD309	89106	PE	BD	560494	EDC
Desmin	RD-301	Purified	BD	550626	Sarcoma
EpCAM	EBA-1	APC	BD	347200	Carcinoma
	EBA-1	PERCP-cy5.5	BD	347199	WT
GD2	14.G2a	Purified	BD	554272	NBL
	14.G2a	FITC	BD	563439	OST
	14.G2a	Alexa fluor 647	BD	562096	
HLA-DR	L243	PERCP-cy5.5	BD	339194	HL
	L243	PB	BD	307624	Monocyte
nuMYOD1	5.8A	Purified	BD	554130	RMS
	5.8A	FITC	Novus Biologicals	NB10056511F	
	polyclonal	FITC	LSBio	LS-C263889	
nuMyogenin	F5D	Purified	BD	556358	RMS
	F5D	PE	BD	563120	
NG2	7.1	PE	Beckman/Coulter	B92429	GCT, BLL
cyOct3/4	40/Oct-3	PE	BD	560186	Carcinoma
smIgM	G20-127	APC	BD	551062	GIB, MBCL
smIgκ κ	polyclonal	PE	Cytognos	CYT-SLPC-50	GIB, MBCL
smIgλ	polyclonal	FITC	Cytognos	12-5825	GIB, MBCL
IgG1	SAG1	FITC	Cytognos	Cyt-IGG1F	NHST

* Results expressed as number of cases and percentage between brackets; [#] 15 no neoplastic tumor mass, 1 non-infiltrated contralateral testis and 1tumor border; [ϕ] 2 cerebrospinal fluid samples.

Table S2. Pattern of antigen expression by tumor cells from different diagnostic categories of pediatric tumors.

**WHO/ICCC-3 Diagnosis (*n*= 113)**	**Immunophenotypic Markers**																	
	CD56	GD2	CD99	CD271	$_{nu}$Myog	$_{nu}$MYOD1	EpCAM	CD9	CD81	CD90	CD44	CD57	CD58	CD10	CD71	CD34	CD105	CD117
Neuroblastoma MFI	38121	14385	364	2183	381	309	209	7144	6880	3887	428	1117	396	145	1400	759	438	1514
(range) (*n* = 38)	(1158-101658)	(1309-72291)	(16-1126)	(30-4328)	(92-1533)	(96-996)	(163-875)	(570-35095)	(1001-43553)	(784-24646)	(104-663)	(45-16305)	(98-2327)	(9-374)	(138-1508)	(51-1944)	(9-189)	(178-3810)
N of positive/infiltrated samples (%)	38/38 (100%)	38/38 (100%)	38/38 (100%)	4/38 (10%)	0/38 (0%)	0/38 (0%)	0/38 (0%)	28/30 (93%)	30/30 (100%)	30/30 (100%)	0/9 (0%)	5/25 (20%)	5/25 (20%)	0/27 (0%)	3/6 (50%)	0/38 (0%)	0/4 (0%)	4/22 (18%)
Ganglioneuroblastoma MFI	56440	69926	439	858	582	417	414	29695	11302	3860	629	458	ND	235	ND	489	ND	352
(range) (*n* = 4)	(10996-92767)	(3227-72400)	(283-591)	(164-914)	(391-444)	(29-788)	(335-1158)	(27919-54739)	(6467-12175)	(3373-5697)	(604-654)	(386-597)		(83-554)		(186-793)		(262-443)
N of positive/infiltrated samples (%)	4/4 (100%)	4/4 (100%)	0/4 (0%)	2/4 (50%)	0/4 (0%)	0/4 (0%)	0/4 (0%)	4/4 (100%)	4/4 (100%)	4/4 (100%)	0/4 (0%)	0/4 (0%)	ND	0/4 (0%)	ND	0/4 (0%)	ND	0/4 (0%)
Ganglioneuroma MFI (*n* = 1)	12803	2735	329	1385	444	386	903	15182	5424	5915	656	684	1572	766	ND	931	ND	1115
N of positive/infiltrated samples (%)	1/1 (100%)	1/1 (100%)	0/1 (0%)	1/1 (100%)	0/1 (0%)	0/1 (0%)	0/1 (0%)	1/1 (100%)	1/1 (100%)	1/1 (100%)	0/1 (0%)	0/1 (0%)	1/1 (100%)	0/1 (0%)	ND	0/1 (0%)	ND	0/1 (0%)
Pheochromocytoma MFI (*n* = 1)	104455	54404	801	1625	183	271	342	9836	11167	31133	331	768	1242	384	ND	955	ND	430
N of positive/infiltrated samples (%)	1/1 (100%)	1/1 (100%)	0/1 (0%)	1/1 (100%)	0/1 (0%)	0/1 (0%)	0/1 (0%)	1/1 (100%)	1/1 (100%)	1/1 (100%)	0/1 0%	1/1 (100%)	1/1 (100%)	0/1 (0%)	ND	0/1 (0%)	ND	0/1 (0%)
Nephroblastoma MFI	19351	571	363	5016	351	387	2266	4643	9150	3001	167	269	868	1771	527	488	2366	207
(range) (*n* = 15)	(3337-49896)	(76-1655)	(94-1703)	(810-18240)	(307-549)	(99-620)	(369-11562)	(797-46240)	(523-25642)	(91-18423)	(64-865)	(87-999)	(63-2526)	(27-4377)	(349-640)	(10-616)	(95-2859)	(76-430)
N of positive/infiltrated samples (%)	15/15 (100%)	0/15 (0%)	0/15 (0%)	15/15 (100%)	0/15 (0%)	0/10 (0%)	13/15 (86%)	15/15 (100%)	15/15 (100%)	5/15 (33%)	0/9 (0%)	0/15 (0%)	0/9 (0%)	3/15 (20%)	0/3 (0%)	0/7 (0%)	2/9 (22%)	0/9 (0%)
Clear cell Sarcoma MFI (*n* = 1)	26025	12560	949	140527	661	ND	856	ND	ND	ND	ND	ND	ND	ND	ND	ND	ND	ND
N of positive/infiltrated samples (%)	1/1 (100%)	1/1 (100%)	0/1 (0%)	1/1 (100%)	0/1 (0%)	ND	0/1 (0%)	ND	ND	ND	ND	ND	ND	ND	ND	ND	ND	ND
Renal cell Carcinoma MFI (*n* = 1)	8342	586	91	10835	ND	310	1846	42	61167	104	201	205	ND	220	935	ND	96	4222
N of positive/infiltrated samples (%)	1/1 (100%)	0/1 (0%)	0/1 (0%)	1/1 (100%)	ND	0/1 (0%)	1/1 (100%)	0/1 (0%)	1/1 (100%)	0/1 (0%)	0/1 0%	0/1 (0%)	ND	0/1 (0%)	1/1 (100%)	ND	0/1 (0%)	1/1 (100%)
Hepatoblastoma MFI (*n* = 1)	4249	1620	683	713	427	748	432	3685	5555	988	1066	2226	ND	914	1985	852	4651	119
N of positive/infiltrated samples (%)	1/1 (100%)	0/1 (0%)	0/1 (0%)	1/1 (100%)	0/1 (0%)	0/1 (0%)	0/1 (0%)	1/1 (100%)	1/1 (100%)	0/1 (0%)	0/1 (0%)	1/1 (100%)	ND	1/1 (100%)	1/1 (100%)	0/1 (0%)	1/1 (100%)	0/1 (0%)

Osteosarcoma MFI	15715	5324	857	3440	636	653	397	2751	8396	3107	734	414	ND	778	2646	347	1603	404
(range) (n = 2)	(15178-16253)	(5101-5547)	(425-1289)	(3127-3753)	(542-703)	(327-979)	(343-452)	(1829-3673)	(4628-12165)	(619-3107)	(351-1117)	(387-442)		(768-788)		(319-375)	(1339-1867)	(383-426)
N of positive/infiltrated samples (%)	2/2 (100%)	2/2 (100%)	0/2 (0%)	2/2 (100%)	0/2 (0%)	0/2 (0%)	0/2 (0%)	2/2 (100%)	2/2 (100%)	1/2 (50%)	0/2 (0%)	0/2 (0%)	ND	0/2 (0%)	1/1 (100%)	0/2 (0%)	2/2 (100%)	0/2 (0%)
Chondrosarcoma MFI (n = 1)	5209	15214	373	190	1168	906	553	20412	4971	10914	ND	440	1347	440	ND	1301	ND	459
N of positive/infiltrated samples (%)	1/1 (100%)	1/1 (100%)	0/1 (0%)	0/1 (0%)	0/1 (0%)	0/1 (0%)	0/1 (0%)	0/1 (0%)	1/1 (100%)	1/1 (100%)	ND	0/1 (0%)	1/1 (100%)	0/1 (0%)	ND	0/1 (0%)	ND	0/1 (0%)
Rhabdomyosarcoma MFI	12275	12456	5272	12473	1940	1157	247	17128	9810	2727	224	2657	224	367	5914	349	100	189
(range) (n = 13)	(1355-52857)	(107-12456)	(984-5272)	(2987-59272)	(835-44399)	(704-61977)	(43-537)	(180-35434)	(3496-22643)	(238-16360)	(59-508)	(40-2657)	(120-1265)	(2-740)	(768-5914)	(230-703)	(100-517)	(19-609)
N of positive/infiltrated samples (%)	13/13 (100%)	1/13 (7%)	1/13 (7%)	13/13 (100%)	9/9 (100%)	7/7 (100%)	0/13 (0%)	5/13 (39%)	13/13 (100%)	5/13 (39%)	0/5 (90%)	1/13 (7%)	0/8 (0%)	0/8 (0%)	1/2 (50%)	0/5 (0%)	0/3 (0%)	0/7 (0%)
Extraosseous Ewing sarcoma MFI (range) (n = 5)	5460 (1206-59635)	2129 (415-2775)	1912 (1283-6280)	4154 (899-23449)	3727 (478-3727)	490 (477-934)	145 (95-762)	4526 (362-12611)	18790 (5732-28988)	4723 (203-6003)	80 (68-148)	1723 (106-1723)	1494 (1383-1605)	2985 (112-2985)	ND	242 (161-1413)	1876 (1654-2098)	7447 (91-13327)
N of positive/infiltrated samples (%)	5/5 (100%)	2/5 (40%)	5/5 (100%)	5/5 (100%)	1/5 (20%)	0/5 (0%)	0/5 (0%)	4/5 (80%)	5/5 (100%)	4/5 (80%)	0/3 (0%)	1/5 (20%)	2/2 (100%)	1/5 (20%)	ND	0/5 (0%)	2/2 (100%)	2/5 (40%)
Malignant nerve sheath tumor (n = 1)	36418	19834	5441	5626	412	504	126	5473	30378	13963	6042	306	ND	8643	4182	ND	106	390
N of positive/infiltrated samples (%)	1/1 (100%)	1/1 (100%)	1/1 (100%)	1/1 (100%)	0/1 (0%)	0/1 (0%)	0/1 (0%)	1/1 (100%)	1/1 (100%)	1/1 (100%)	1/1 (100%)	0/1 (0%)	ND	1/1 (100%)	1/1 (100%)	ND	0/1 (0%)	0/1 (0%)
Germ cell tumor MFI	11192	678	419	1862	214	271	3286	2726	6743	2441	294	369	2706	250	472	638	448	182
(range) (n = 13)	(64-49637)	(166-1361)	(38-1088)	(252-10730)	(80-936)	(125-1092)	(121-6734)	(1323-18863)	(1532-19227)	(440-7349)	(167-707)	(92-780)	(747-4025)	(51-574)	(149-620)	(23-1586)	(113-912)	(74-269)
N of positive/infiltrated samples (%)	5/13 (39%)	0/13 (0%)	0/13 (0%)	5/13 (39%)	0/13 (0%)	0/7 (0%)	4/13 (30%)	13/13 (100%)	13/13 (100%)	5/13 (39%)	0/5 (0%)	0/8 (0%)	2/4 (50%)	0/9 (0%)	0/4 (0%)	0/8 (0%)	0/4 (0%)	0/4 (0%)
Nasopharyngeal carcinoma MFI (range) (n = 2)	10460 (23-10460)	569 (274-865)	496 (304-688)	488 (426-550)	807 (514-1100)	777 (393-1162)	23341 (1352-45330)	6111 (5833-6390)	4225 (3759-4691)	1706 (91-1706)	29	434 (82-787)	2122 (608-2122)	367 (111-623)	ND	129 (123-135)	ND	167
N of positive/infiltrated samples (%)	1/2 (50%)	0/2 (0%)	0/2 (0%)	0/2 (0%)	0/2 (0%)	0/2 (0%)	2/2 (100%)	2/2 (100%)	2/2 (100%)	1/2 (50%)	0/1 (0%)	0/2 (0%)	1/2 (50%)	0/2 (0%)	ND	0/2 (0%)	ND	0/1 (0%)
Adrenal carcinoma MFI	3167	747	368	1300	176	277	1012	1352	6054	9284	324	200	ND	530	ND	546	592	134
(range) (n = 4)	(143-5003)	(682-1879)	(330-623)	(360-1300)	(118-198)	(118-436)	(305-1376)	(682-1533)	(89-24209)	(52-9284)	(282-367)	(82-319)		(115-753)		(213-902)	(539-645)	(120-349)
N of positive/infiltrated samples tested (%)	2/4 (50%)	0/4 (0%)	0/4 (0%)	1/4 (25%)	0/4 (0%)	0/2 (0%)	2/4 (50%)	2/4 (50%)	2/4 (50%)	1/2 (50%)	0/2 (0%)	0/2 (0%)	ND	0/3 (0%)	ND	0/3 (0%)	0/2 (0%)	0/2 (0%)

Undifferentiated malignant neoplasm MFI (range) (*n* = 2)	30259 (16420-44098)	3449	877 (257-877)	22946 (3967-41926)	871	440	345 (208-482)	4154 (519-7789)	8824 (4860-12789)	515 (402-629)	260	3813 (241-3813)	793	2417 (213-2417)	477	638	208	163
N of positive/infiltrated samples tested (%)	2/2 (100%)	1/1 (100%)	1/2 (50%)	2/2 (100%)	0/1 (0%)	0/1 (0%)	0/2 (0%)	2/2 (100%)	2/2 (100%)	0/2 (0%)	0/1 (0%)	1/2 (50%)	0/1 (0%)	1/2 (50%)	0/1 (0%)	0/1 (0%)	0/1 (0%)	0/1 (0%)
Pseudopapilary pancreatic solid tumor MFI (*n* = 1)	18896	372	689	8332	2568	560	314	32947	11701	11	492	307	ND	3603	659	482	98	ND
N of positive/infiltrated samples (%)	1/1 (100%)	0/1 (0%)	0/1 (0%)	1/1 (100%)	0/1 (0%)	0/1 (0%)	0/1 (0%)	1/1 (100%)	1/1 (100%)	0/1 (0%)	0/1 (0%)	0/1 (0%)	ND	1/1 (100%)	0/1 (0%)	0/1 (0%)	0/1 (0%)	ND
Mesoblastic nephroma MFI (range) (*n* = 2)	121 (84-326)	563 (425-701)	478 (343-614)	1447 (1287-1607)	622	332	15232 (9985-20480)	10347 (5504-15190)	11140 (5526-16774)	35972 (26521-45424)	390 (352-428)	476 (116-837)	3266	66516 (64150-68882)	ND	303 (177-430)	969	414 (384-444)
N of positive/infiltrated samples (%)	0/2 (0%)	0/2 (0%)	0/2 (0%)	2/2 (100%)	0/1 (0%)	0/1 (0%)	2/2 (100%)	2/2 (100%)	2/2 (100%)	2/2 (100%)	0/2 (0%)	0/2 (0%)	1/1 (100%)	2/2 (100%)	ND	0/2 (0%)	0/1 (0%)	0/2 (0%)
Cystic nephroma MFI (*n* = 1)	31	294	375	733	131	292	1484	6442	14631	302	426	239	1415	16998	768	ND	ND	ND
N of positive/infiltrated samples (%)	0/1 (0%)	0/1 (0%)	0/1 (0%)	0/1 (0%)	0/1 (0%)	0/1 (0%)	1/1 (100%)	1/1 (100%)	1/1 (100%)	0/1 (0%)	0/1 (0%)	0/1 (0%)	1/1 (100%)	1/1 (100%)	0/1 (0%)	ND	ND	ND
Hemangioma MFI (*n* = 1)	569	121	692	251	165	88	612	435	1171	48285	652	ND	8498	6576	ND	9336	ND	331
N of positive/infiltrated samples (%)	0/1 (0%)	0/1 (0%)	0/1 (0%)	0/1 (0%)	0/1 (0%)	0/1 (0%)	0/1 (0%)	0/1 (0%)	1/1 (100%)	1/1 (100%)	0/1 (0%)	ND	1/1 (100%)	1/1 (100%)	ND	1/1 (100%)	ND	0/1 (0%)
Hemangiopericytoma MFI (*n* = 1)	1088	1074	503	167	224	133	371	2011	1443	374	ND	264	2561	449	ND	6082	ND	791
N of positive/infiltrated samples (%)	0/1 (0%)	0/1 (0%)	0/1 (0%)	0/1 (0%)	0/1 (0%)	0/1 (0%)	0/1 (0%)	1/1 (100%)	1/1 (100%)	0/1 (0%)	ND	0/1 (0%)	1/1 (100%)	0/1 (0%)	ND	1/1 (100%)	ND	0/1 (0%)
Neurofibroma MFI (*n* = 1)	18954	4758	346	62897	840	739	518	2174	14338	522	234	222	ND	4757	401	9150	888	234
N of positive/infiltrated samples (%)	1/1 (100%)	1/1 (100%)	0/1 (0%)	1/1 (100%)	0/1 (0%)	0/1 (0%)	0/1 (0%)	1/1 (100%)	1/1 (100%)	0/1 (0%)	0/1 (0%)	0/1 (0%)	ND	1/1 (100%)	0/1 (0%)	1/1 (100%)	0/1 (0%)	0/1 (0%)
Benign cutaneous fibrohistiocytic tumor MFI (*n* = 1)	188	256	631	41	ND	439	144	3329	2914	20855	ND	244	727	46952	ND	350	ND	162
N of positive/infiltrated samples (%)	0/1 (0%)	0/1 (0%)	0/1 (0%)	0/1 (0%)	ND	0/1 (0%)	0/1 (0%)	1/1 (100%)	1/1 (100%)	1/1 (100%)	ND	0/1 (0%)	0/1 (0%)	1/1 (100%)	ND	0/1 (0%)	ND	0/1 (0%)

MFI: mean fluorescence intensity (arbitrary units scaled from 0 to 101658 fluorescence channels).

Table S3. Demographic and clinical features of pediatric patients (*n* = 296) and their samples (*n* = 476) analyzed in this study.

Parameter	Patient Distribution (*n* = 296) *	N. of Infiltrated/Total Samples (%) (*n* = 476) *
Age at diagnosis (Years) *	7.3 ± 4.8	
Gender (Male-Female)	181-115	
Underling disease		
Reactive	72 (24%)	0/95 (0%)
Benign neoplasm	8 (3%)	8/9 (89%)
Malignant neoplasm	216 (73%)	228/372 (61%)
-Localized tumor	127 (59%)	106/204 (52%)
-Metastatic tumor	89 (41%)	122/168 (73%)
Type of sample	Primary site (*n* = 296)	Sample site (*n* = 476)
Tumor mass	240 (81%)	114/139 (82%)
-abdominal	94 (32%)	48/58 (83%)
-thoracic	61 (21%)	24/28 (86%)
-head and neck	32 (11%)	12/15 (80%)
-pelvic	31 (10%)	15/15 (100%)
-soft tissue mass	7 (2%)	3/6 (50%)
-thoracoabdominal	5 (1.7%)	4/4 (100%)
-bone	5 (1.7%)	2/3 (67%)
-testis	3 (1%)	5/6 (83%)
-tonsil	1 (0.3%)	0/2 (0%)
-CNS	1 (0.3%)	0 (0%)
-peritoneal metastasis	NP [Φ]	1/1 (100%)
-pericardial biopsy	NP [Φ]	0/1 (0%)
Lymph node	50 (17%)	32/86 (37%)
Body effusion	NA	28/55 (51%)
BM	6 (2%)	41/138 (30%)
Peripheral blood	NA	13/58 (22%)
ICCC-3/ WHO diagnosis	Patient (*n* = 296)	Sample (*n* = 476)
Leukemias, myeloproliferative and myelodysplastic diseases	42 (14%)	56/82 (68%)
Lymphomas and reticuloendothelial neoplasms	69 (23%)	62/112 (55%)
Neuroblastoma and other peripheral nervous cell tumors	45 (15%)	44/79 (56%) [&]
Renal tumors	17 (5.7%)	17/22 (77%)
Hepatic tumors	2 (0.7%)	2/2 (100%)
Malignant bone tumors	5 (1.7%)	4/8 (50%)
Soft tissue and other extraosseous sarcomas	16 (5.4%)	20/36 (55%) [#]
Germ cell tumors	10 (3.3%)	13/16 (81%)
Malignant epithelial neoplasms	6 (2%)	7/9 (78%)
CNS miscellaneous intracranial and intraspinal neoplasms	1 (0.3%)	0/3 (0%)
Other rare and unspecified malignant neoplasms	3 (1%)	3/3 (100%)
Benign neoplasms	8 (2.7%)	8/9 (89%)
Inflammatory/reactive diseases	72 (24%)	0/95 (0%)
Time of analysis		
At diagnosis	265 (90%)	200/423 (47%)
On chemotherapy	17 (6%)	12/31 (39%)
At relapse	14 (4%)	16/22 (73%)
Total	296 (100%)	236/476 (49%)

* Results expressed as number of patients and percentage between brackets or as * median ± one standard deviation; & 3 peripheral blood with circulating tumor cells; # 1 peripheral blood with circulating tumor cells; * 2 samples of cerebrospinal fluid; * NP: not primary site (metastatic site, samples received for stage procedures together with pelvic tumor and thoracic tumor, respectively).

Table S4. EuroFlow panels of antibody combinations for diagnostic classification of B-cell precursor and T-cell acute lymphoblastic leukemia/lymphoma and acute myeloid leukemia following the acute leukemia orientation tube (ALOT).

**EuroFlow Antibody Panel**	**Fluorochrome Conjugated Reagents-Hematopoietic Tumors**							
	**PB**	**PO**	**FITC**	**PE**	**PerCPCy5.5**	**PECy7**	**APC**	**APCH7**
BCP-ALL (*n* = 38)	CD20	CD45	CD58	CD66c	CD34	CD19	CD10	CD38
	smIgκ	CD45	cyIgμ	CD33	CD34	CD19	smIgM + CD117	smIgλ
	CD9	CD45	nuTdt	CD13	CD34	CD19	CD22	CD24
	CD21	CD45	CD15 + CD65	NG2	CD34	CD19	CD123	CD81
T-ALL (*n* = 27)	cyCD3	CD45	nuTdT	CD99	CD5	CD10	CD1a	smCD3
	cyCD3	CD45	CD2	CD117	CD4	CD8	CD7	smCD3
	cyCD3	CD45	TCRγδ	TCRαβ	CD33	CD56	cyTCRβ	smCD3
	cyCD3	CD45	CD44	CD13	HLADR	CD45RA	CD123	smCD3
AML/MDS (*n* = 8)	HLADR	CD45	CD16	CD13	CD34	CD117	CD11b	CD10
	HLADR	CD45	CD35	CD64	CD34	CD117	CD300e	CD14
	HLADR	CD45	CD36	CD105	CD34	CD117	CD33	CD71
	HLADR	CD45	nuTdT	CD56	CD34	CD117	CD7	CD19
	HLADR	CD45	CD15	NG2	CD34	CD117	CD22	CD38
	HLADR	CD45	CD61 + CD42a	CD203c	CD34	CD117	CD123	CD4
	HLADR	CD45	CD41	CD25	CD34	CD117	CD42b	CD9

Abbreviations (alphabetical order): APC: allophycocyanin; AML/MDS: acute myeloid leukemia/myelodysplastic syndrome; APCH7: allophycocyanin-hilite 7; BCP-ALL: B-cell precursor acute lymphoblastic leukemia; cy: cytoplasmic; FITC: fluorescein isothiocyanate; nu: nuclear; Ig: immunoglobulin; PerCPCy5.5: peridinin-chlorophyll protein-complex cyanin 5.5; PE: phycoerythrin; PE-Cy7: PE-cyanin 7; sm: surface membrane; T-ALL: T-cell acute lymphoblastic leukemia; TCR: T-cell receptor; Tdt: Terminal Deoxynucleotidyl Transferase.

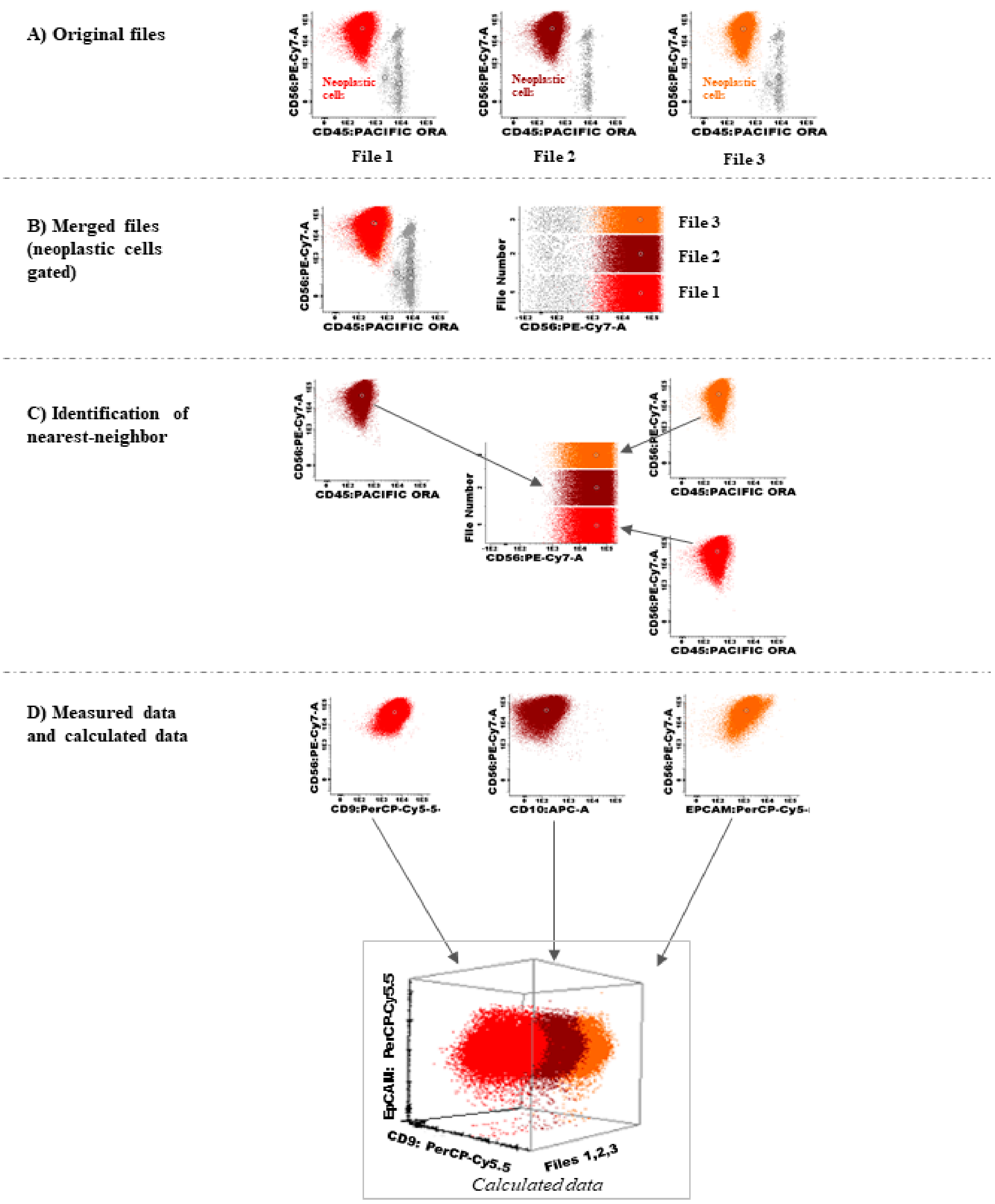

Figure S1. Schematic representation of the data file merge and calculation procedure used for construction of the database. (**A**) Multiple data files corresponding to different aliquots of an individual tumor sample containing data about a set of common "backbone markers" (e.g., SSC, FSC, CD45 and CD56) and other markers stained specifically in each individual aliquot of the sample were selected and tumor cells color gated; (**B**) MFC data files of neoplastic cells gated on the common backbone parameters were merged into a single data file; (**C**) after selection of neoplastic cells in each data file, for each neoplastic cellular event present in one data file (e.g., calculated dot in data file 1), the nearest neighbor (e.g., circled dot in data file 3) was identified in each of the other data files (e.g., file 1, red; file 2, brown; file 3, orange); (**D**) the values obtained for those parameters actually measured in each individual event from one data file were assigned to the nearest neighbor cellular event from data files in which that parameter had not been actually measured, to generate a final data file that contained data for all parameters studied for every individual tumor cell measured in all aliquots of the (same) tumor sample.
